# From Mineral Surfaces to Peptides: Hydroxyapatite-Based Platforms for Surface-Mediated Prebiotic Synthesis

**DOI:** 10.3390/ijms27136008

**Published:** 2026-07-04

**Authors:** Jordi Puiggalí

**Affiliations:** Departament de Enginyeria Química, Escola d’Enginyeria de Barcelona Est, Universitat Politècnica de Catalunya, Av. Eduard Maristany 10-14, 08019 Barcelona, Spain; jordi.puiggali@upc.edu

**Keywords:** prebiotic chemistry, origin of life, hydroxyapatite, mineral surfaces, peptide bond formation, amino acids, heterogeneous catalysis, surface-mediated synthesis, nitrogen fixation, mineral–organic interfaces

## Abstract

The formation of peptide bonds under prebiotic conditions represents a major challenge due to both thermodynamic and kinetic constraints, particularly in aqueous environments where condensation reactions are disfavored. Mineral surfaces have long been proposed as key contributors to overcoming these limitations by providing structured and reactive interfaces that promote molecular organization and facilitate chemical transformations. In this context, hydroxyapatite emerges as a particularly promising system due to its structural versatility, surface heterogeneity, and ability to interact with a wide range of organic molecules. Its capacity to support adsorption, interfacial organization, and dynamic interactions makes it a promising platform for surface-mediated prebiotic chemistry. Furthermore, the incorporation of metal centers, especially zirconium-based species, introduces additional catalytic functionalities that can enhance bond activation and enable cooperative reaction mechanisms. The combination of mineral surfaces and metal-based catalysis thus provides a framework for understanding how complex chemical processes could have emerged under prebiotic conditions. Particular attention is given to hybrid hydroxyapatite–zirconium systems as multifunctional catalytic platforms integrating adsorption, activation, and spatial organization. Finally, the role of dynamic environmental regimes, including gradients, cyclic processes, and non-equilibrium conditions, is considered as a critical factor in sustaining chemical reactivity and promoting increasing levels of molecular complexity. Together, these elements support a scenario in which surface-mediated processes played a central role in the emergence of peptide-like structures and early protometabolic systems.

## 1. Introduction

The origin of life remains one of the most fundamental and unresolved questions in science, motivating extensive efforts to identify plausible chemical pathways leading from simple inorganic precursors to the first functional biomolecules. Early conceptual frameworks, notably those proposed by Alexander Oparin, emphasized the gradual chemical evolution of organic matter under primitive Earth conditions, laying the foundation for modern prebiotic chemistry [1]. In this context, prebiotic chemistry refers to the set of abiotic chemical processes that may have operated on the early Earth before the emergence of biological systems, leading from simple inorganic and organic precursors to increasingly complex molecular structures.

Subsequent experimental and theoretical developments significantly advanced this field. Notably, the work of Joan Oró demonstrated the abiotic formation of biologically relevant molecules such as adenine from simple precursors [2], while the contributions of Leslie Orgel established key principles for prebiotic synthesis and molecular self-organization [3]. Together, these studies provided a framework in which solution-phase chemistry became the dominant paradigm for exploring the origin of biomolecular complexity.

In addition to amino acids, considerable attention has been devoted to the prebiotic synthesis of nitrogenous bases, which are essential components of nucleic acids and central to hypotheses involving early self-replicating systems. However, the formation of nucleobases under plausible prebiotic conditions presents significant chemical challenges, including the need for specific reaction pathways, controlled environments, and relatively complex precursor molecules [4,5]. While notable advances have been made, the efficiency and generality of such processes remain subjects of ongoing debate.

In contrast, amino acids are generally considered more accessible under a wider range of prebiotic conditions and can participate in relatively simpler reaction networks. Moreover, peptide formation represents a critical step toward functional molecular systems, as short peptides may exhibit catalytic or structural properties even in the absence of genetic replication [6]. For these reasons, the present work focuses on amino acid-based pathways and peptide formation, particularly in the context of surface-mediated processes.

**Figure 1:**
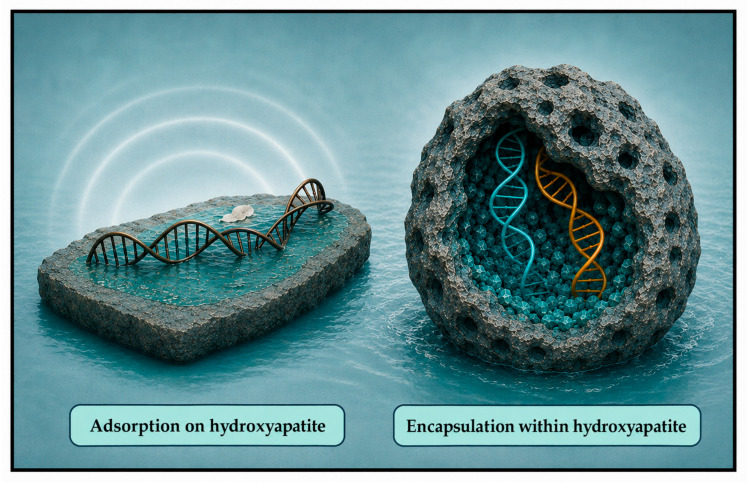
Conceptual schematic illustration of DNA interactions with hydroxyapatite under conditions relevant to prebiotic environments. **Left**: adsorption of DNA onto hydroxyapatite surfaces through interactions between the phosphate backbone and surface calcium sites, promoting molecular stabilization and organization. **Right**: encapsulation of DNA within hydroxyapatite during mineral growth, leading to the formation of hybrid mineral–organic structures with enhanced resistance to chemical degradation. The illustration provides a conceptual interpretation of mineral–biomolecule interactions inspired by mechanisms and graphical representations discussed in our previously reported studies [10].

Mineral systems have increasingly been recognized as key components in prebiotic chemistry, providing not only structural support but also active interfaces for molecular adsorption and transformation. Among these, hydroxyapatite has attracted particular attention due to its capacity to interact with a wide range of ionic and molecular species, as well as its ability to incorporate substitutions and defects that may influence its physicochemical properties [7,8,9]. In addition to its relevance in biomineralization processes, hydroxyapatite has been shown to strongly interact with biomolecules such as DNA, promoting their stabilization and protection under harsh conditions while preserving their structural integrity [10,11,12]. These interactions include both surface adsorption, mediated by the affinity between phosphate groups and calcium sites, and encapsulation processes occurring during mineral growth. A schematic representation of these concepts is shown in Figure 1.

While these approaches have significantly advanced our understanding of prebiotic synthesis, they also highlight a fundamental challenge: the translation of plausible chemical pathways into efficient and sustained reaction systems under realistic prebiotic conditions. In particular, the formation and subsequent transformation of small organic molecules, including amino acids, require not only appropriate precursors but also environments that can promote reactivity beyond what is typically observed in homogeneous solutions. Prebiotic environments were likely characterized by low concentrations of reactants, leading to severe dilution effects that hinder efficient molecular interactions. In addition, the absence of structural organization in homogeneous systems limits the probability of productive collisions and reduces the efficiency of condensation reactions, such as peptide bond formation, which are thermodynamically and kinetically unfavorable in aqueous media [3,4,5].

**Figure 2:**
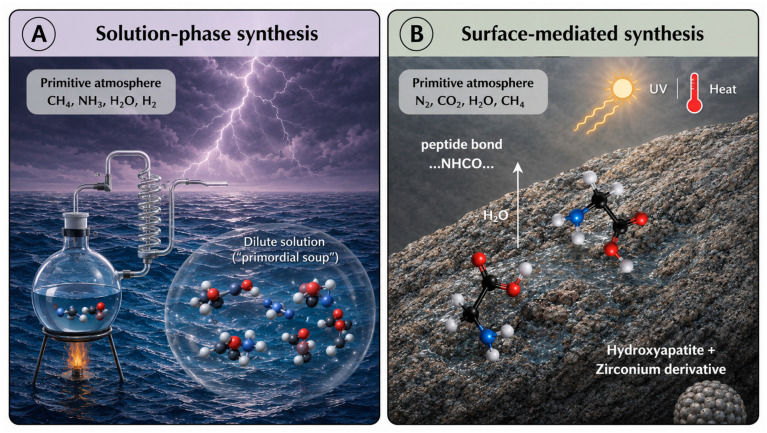
Comparison between classical solution-phase prebiotic synthesis and surface-mediated pathways. (**A**) In the classical Miller–Urey-type model, chemical evolution proceeds in homogeneous aqueous environments following gas-phase activation (e.g., electrical discharges), leading to the formation of organic molecules in dilute and poorly organized systems (“primordial soup”). (**B**) In contrast, surface-mediated synthesis on mineral substrates (e.g., hydroxyapatite-based systems) enables adsorption and concentration of simple atmospheric molecules (N_2_, CO_2_, H_2_O, CH_4_), while external energy inputs such as ultraviolet radiation and heat may promote localized and more selective chemical transformations. This comparison highlights the potential advantages of heterogeneous environments in overcoming limitations associated with dilution, lack of organization, and low reaction efficiency in solution-phase models.

In contrast, mineral surfaces provide distinct physicochemical environments that may overcome several of these constraints. Adsorption onto solid interfaces can result in local concentration of reactants, reduced dimensionality, and enhanced molecular orientation, thereby facilitating chemical transformations that are unlikely to occur in bulk solution. Furthermore, surface-mediated processes can generate microenvironments characterized by confinement, gradients, and transient dehydration conditions, all of which are highly relevant for condensation chemistry and polymer formation [6,13,14,15]. This conceptual contrast between homogeneous and surface-mediated pathways is summarized in Figure 2.

**Figure 3:**
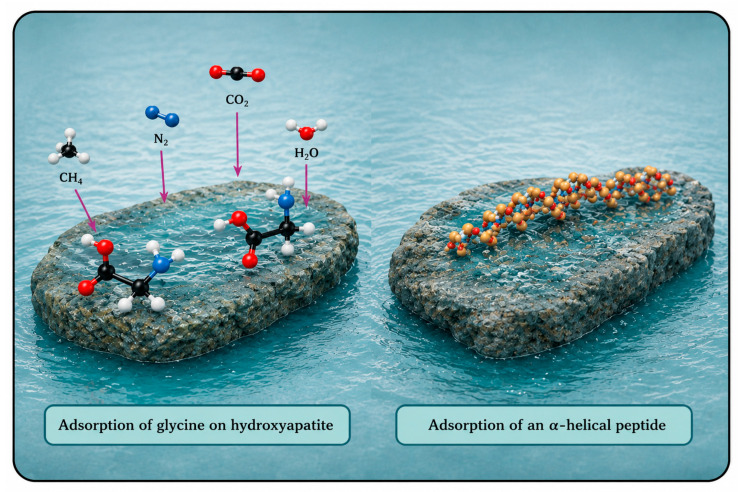
Conceptual illustration of surface-mediated prebiotic synthesis on hydroxyapatite-based materials. **Left**: formation and adsorption of glycine (NH_2_–CH_2_–COOH) from simple precursors (N_2_, CO_2_, and H_2_O) on mineral surfaces, highlighting the role of adsorption and activation in amino acid synthesis. **Right**: adsorption and organization of a short α-helical peptide on the mineral surface, illustrating the potential emergence of secondary structure in surface-confined environments. The illustration represents a conceptual interpretation of mineral-catalyzed molecular evolution.

The interaction of simple atmospheric molecules such as N_2_, CO_2_, H_2_O, and CH_4_ with mineral surfaces under external energy inputs (e.g., thermal gradients or ultraviolet radiation) may enable chemical transformations that are otherwise kinetically hindered. In this context, recent studies have demonstrated that hybrid mineral systems can promote the direct transformation of simple gaseous mixtures into biologically relevant compounds under relatively mild conditions. A representative example is the catalytic fixation of molecular nitrogen and carbon dioxide into amino acids using hydroxyapatite-based materials under UV irradiation, which has been reported to yield glycine and alanine [16]. A conceptual illustration of these surface-mediated processes is presented in Figure 3. These results support the view that surface-mediated processes combining adsorption, energy input, and catalytic activation may provide viable pathways for overcoming the intrinsic inertness of small molecules such as N_2_.

Beyond adsorption-driven processes, increasing attention has been directed toward the catalytic contribution of metal centers in prebiotic systems. Metal ions and metal-containing materials can act as Lewis acid sites, promoting activation of functional groups and lowering the energetic barriers associated with bond formation. For instance, zirconium-based systems exhibit notable catalytic properties in condensation reactions, including amide bond formation, suggesting a potential role in peptide synthesis under prebiotic conditions [17,18,19].

The combination of structured mineral surfaces and catalytically active metal centers leads naturally to the concept of hybrid systems, in which adsorption, organization, and activation processes occur in a cooperative manner. In such systems, hydroxyapatite may provide a platform for molecular concentration and organization, while zirconium species may act as active catalytic sites; together, they enable reaction pathways that are inefficient or inaccessible in homogeneous environments.

In this review, we examine the potential role of mineral surfaces, with particular emphasis on hydroxyapatite-based systems, in addressing key challenges in prebiotic chemistry, including nitrogen availability, amino acid stabilization, and peptide bond formation. Special attention is given to the interplay between surface-mediated processes and catalytic activation, as well as to the possible importance of dynamic, non-equilibrium conditions in promoting complex chemical transformations.

Rather than focusing on a single mechanistic pathway, this work aims to provide an integrative perspective in which adsorption, molecular organization, and catalytic activation are considered as interconnected processes occurring at mineral–organic interfaces. Within this framework, hybrid systems combining structured mineral substrates and catalytically active centers are discussed as potentially relevant environments for enabling chemical transformations that are inefficient in homogeneous conditions. By bringing together insights from mineral chemistry, catalysis, and prebiotic synthesis, this review highlights underexplored pathways that may have contributed to the emergence of early peptides and functional molecular systems.

## 2. The Prebiotic Nitrogen Problem

Nitrogen is an essential element in all known forms of life, being a fundamental component of amino acids, nucleotides, and other biomolecules. However, the availability of chemically reactive nitrogen species on the early Earth remains a central challenge in prebiotic chemistry. Although molecular nitrogen (N_2_) was likely abundant in the primitive atmosphere, it is characterized by exceptional thermodynamic stability due to the strong triple bond (N≡N), with a bond dissociation energy of approximately 941 kJ·mol^−1^ [20]. As a result, N_2_ is generally considered chemically inert under ambient conditions, making its activation a critical bottleneck for the synthesis of nitrogen-containing organic compounds.

In modern industrial chemistry, nitrogen fixation is achieved primarily through the Haber–Bosch process, which converts N_2_ into ammonia (NH_3_) under highly demanding conditions, typically involving temperatures of 400–500 °C, pressures of 150–300 bar, and the use of iron-based catalysts [21]. While this process is highly efficient at industrial scale, it is energetically intensive and relies on conditions that are unlikely to have been sustained in most prebiotic environments. The thermodynamic and kinetic barriers associated with nitrogen activation thus raise fundamental questions about how reactive nitrogen species could have been generated on the early Earth.

Several alternative pathways for abiotic nitrogen fixation have been proposed, including high-energy processes such as lightning discharges, ultraviolet radiation, and impact-driven shock chemistry [22,23,24]. These mechanisms can lead to the formation of nitrogen oxides (NOx), ammonia, or other reactive intermediates, which may subsequently participate in prebiotic synthesis pathways. However, such processes are often sporadic and spatially heterogeneous, potentially limiting the continuous availability of reactive nitrogen species. Recent advances in transition metal chemistry have expanded the conceptual framework for dinitrogen activation beyond its classical reduction to ammonia. In particular, homogeneous catalytic systems have demonstrated that N_2_ can be directly incorporated into organic molecules through metal-mediated C–N bond formation pathways. These processes involve coordination of dinitrogen to metal centers followed by electronic activation via π-backdonation, which weakens the N≡N bond and enables subsequent functionalization steps [25]. Although such systems are typically studied under controlled laboratory conditions, they provide important mechanistic insights into alternative routes for nitrogen activation. In a prebiotic context, these findings suggest that mineral surfaces containing metal centers could, in principle, facilitate analogous activation processes, expanding the range of plausible pathways for the generation of reactive nitrogen species.

In addition to atmospheric processes, geochemical environments such as hydrothermal systems have been suggested as potential sites for nitrogen activation. Interactions between reduced minerals and nitrogen-containing species may facilitate partial reduction or transformation of nitrogen under specific conditions [26]. Nevertheless, the efficiency and general relevance of such processes remain under debate, particularly with respect to their capacity to sustain the formation of amino acids and other key biomolecules.

Recent work has also challenged the widespread assumption that hydrocarbons detected in serpentinization-associated systems are predominantly of abiotic origin. Taguchi et al. [27] showed, using clumped isotopologue analyses combined with molecular and isotopic signatures, that a significant fraction of organic compounds in Mariana forearc serpentinite mud volcanoes is derived from thermogenic alteration of subducted organic matter rather than in situ abiotic synthesis. These findings highlight the importance of applying multiple geochemical proxies when assessing the origin of organic molecules, as reliance on bulk isotopic compositions alone may lead to an overestimation of abiotic contributions in geofluid systems.

An alternative and increasingly explored possibility is that mineral surfaces may have played a direct and active role in nitrogen activation. Adsorption of small molecules such as N_2_, CO_2_, H_2_O, and CH_4_ onto reactive surfaces can modify their electronic structure, promote local concentration, and favor specific molecular orientations. These effects may significantly lower activation barriers, enabling chemical transformations that are otherwise inaccessible in homogeneous environments. In parallel, recent theoretical studies have explored alternative pathways for direct C–N bond formation involving simple atmospheric molecules such as N_2_ and CO_2_. Density functional theory calculations have shown that, under appropriate activation conditions, the coupling between molecular nitrogen and carbon dioxide can become thermodynamically feasible, leading to the formation of nitrogen–carbon bonded intermediates. These studies highlight the importance of electronic activation mechanisms, including charge transfer and orbital interactions, in overcoming the intrinsic inertness of N_2_. Although such systems often rely on model catalysts that are not strictly prebiotic, they provide valuable mechanistic insight into how simple molecules could be directly incorporated into organic frameworks under surface-mediated conditions [28].

Importantly, surface-mediated processes are likely to have operated under non-equilibrium conditions, where external energy inputs such as thermal gradients or ultraviolet radiation could drive chemical reactivity. In such scenarios, the combination of adsorption and energy input may facilitate the activation of otherwise inert molecules, including molecular nitrogen. Recent experimental evidence is consistent with the idea that under these conditions, simple gaseous mixtures can be transformed into amino acids when appropriate catalytic surfaces are present [16].

These observations raise the question of whether alternative catalytic environments, distinct from classical homogeneous or high-pressure systems, could have enabled nitrogen activation under prebiotic conditions. For instance, hybrid systems combining structured mineral surfaces and catalytically active centers may provide unique environments in which adsorption, activation, and transformation processes occur in a cooperative manner. Such systems may not only facilitate the activation of nitrogen-containing species, but also their subsequent incorporation into more complex organic molecules.

The difficulty in activating nitrogen also has important implications for the synthesis of more complex nitrogen-containing compounds, such as nucleobases. These molecules typically require more elaborate reaction pathways and controlled conditions, further emphasizing the importance of identifying efficient and plausible mechanisms for nitrogen activation in prebiotic environments. In this context, the study of surface-mediated processes emerges as a promising avenue for bridging the gap between simple inorganic precursors and the formation of biologically relevant molecules.

## 3. Amino Acid Formation in Prebiotic Environments

The formation of amino acids under prebiotic conditions has been extensively investigated and is generally considered more accessible than the synthesis of more complex nitrogen-containing biomolecules. Classical experiments have demonstrated that amino acids can be generated from simple precursors under a variety of conditions, including electrical discharges, such as those demonstrated in the classic Miller–Urey experiments, as well as Strecker-type synthesis pathways and other aqueous-phase reactions [29,30,31]. These findings support the idea that amino acids could have been readily available on the early Earth.

Despite these advances, several limitations remain. Many of the proposed synthetic pathways rely on specific conditions, such as controlled atmospheres or high-energy inputs, which may not have been uniformly present in prebiotic environments. In addition, the yields of amino acids are often low, and their stability can be compromised by competing reactions, hydrolysis, or degradation processes that may limit their accumulation under prebiotic conditions [32,33]. These factors raise important questions about the persistence of amino acids in sufficient concentrations to enable further chemical evolution.

From a chemical perspective, the formation of amino acids typically requires the incorporation of nitrogen into organic frameworks, which remains closely linked to the challenges associated with nitrogen activation discussed in the previous section. While reduced nitrogen species such as ammonia can readily participate in amino acid synthesis, the direct involvement of molecular nitrogen remains significantly more difficult due to its inherent inertness. This further emphasizes the importance of identifying environments capable of facilitating both nitrogen activation and subsequent incorporation into organic molecules. In this context, recent advances in catalytic systems have demonstrated that glycine can be selectively formed through C–N coupling reactions involving carbonyl intermediates and reduced nitrogen species. For example, electrocatalytic and photocatalytic approaches have shown that intermediates such as glyoxylic acid or glycolaldehyde can react with nitrogen-containing species (e.g., NH_3_ or NH_2_OH) to yield glycine with significant selectivity. These studies emphasize the critical role of surface-mediated stabilization of reactive intermediates in directing product formation and preventing overoxidation pathways. Although these systems are based on engineered catalysts, they provide important conceptual analogues for understanding how mineral surfaces in prebiotic environments could promote selective amino acid synthesis [34].

In addition to their formation, the spatial and chemical organization of amino acids represents a critical step toward increasing molecular complexity. In homogeneous aqueous systems, amino acids are subject to dilution and random distribution, which limits their ability to undergo further reactions such as condensation. This limitation is particularly relevant for peptide bond formation, which is both thermodynamically and kinetically unfavorable in bulk aqueous environments.

Mineral surfaces offer a promising solution to these constraints by providing environments that can promote adsorption, concentration, and orientation of amino acids. Experimental and theoretical studies have shown that amino acids can strongly interact with a variety of mineral surfaces, including clays, oxides, and phosphate-based materials, leading to enhanced local concentrations and modified reactivity [6,7,8,9,14,35]. Such interactions may facilitate the formation of ordered assemblies and increase the probability of productive chemical transformations.

In this context, hydroxyapatite represents a particularly interesting system due to its ability to interact with both ionic species and organic molecules, including amino acids and more complex biomolecular structures. Its surface properties, combined with its structural adaptability, suggest that it may play a dual role as both an adsorptive platform and a mediator of chemical reactivity. These features are especially relevant when considering the transition from simple molecular precursors to more complex systems involving peptide bond formation.

Moreover, the presence of catalytically active centers associated with mineral surfaces may further enhance the reactivity of adsorbed species. In particular, the incorporation of metal centers capable of acting as Lewis acids may promote activation of functional groups, facilitating condensation reactions under relatively mild conditions. This is of particular importance in the context of peptide bond formation, where activation of the carboxyl group is a key step.

Recent developments suggest that surface-mediated systems combining adsorption, catalytic activation, and external energy inputs may provide efficient pathways for amino acid formation and transformation under prebiotic conditions. In such systems, the interaction of simple gaseous molecules with reactive surfaces may lead not only to the formation of amino acids, but also to their subsequent organization and evolution toward more complex molecular assemblies.

These considerations reinforce the idea that amino acid formation cannot be fully understood in isolation, but must be considered within the broader context of heterogeneous environments, where adsorption, activation, and reaction processes are intrinsically coupled. This perspective provides a conceptual bridge between the formation of simple organic molecules and the emergence of more complex systems, including peptides and early functional biomolecules.

## 4. Mineral Surfaces in Prebiotic Chemistry

Mineral surfaces have long been considered key components in prebiotic chemistry, providing not only structural support but also active environments capable of promoting chemical reactions. Early hypotheses proposed that minerals could have acted as scaffolds for molecular organization, facilitating the transition from simple molecules to more complex chemical systems [6,35,36,37]. Over time, this view has evolved toward a more dynamic perspective in which mineral surfaces actively participate in adsorption, catalysis, and molecular transformation processes.

One of the primary roles of mineral surfaces in prebiotic environments is the adsorption of small molecules and organic compounds. Adsorption can lead to significant local concentration of reactants, effectively overcoming the dilution problem inherent to aqueous environments [8,14]. In addition, surface interactions can impose specific orientations on adsorbed molecules, reducing configurational entropy and increasing the probability of productive chemical interactions. These effects are particularly relevant for reactions involving condensation or bond formation, which are otherwise disfavored in bulk solution.

### 4.1. Clay Minerals and Nanoconfined Environments

Among the different classes of minerals investigated in prebiotic chemistry, clay minerals have received particular attention due to their high surface area, layered structure, and ion-exchange capacity. Clay minerals such as montmorillonite have been shown to catalyze the formation of RNA oligomers and other polymeric structures, highlighting their potential role in early molecular evolution [7,38]. These materials provide interlayer spaces and charged surfaces that can adsorb and organize organic molecules, thereby facilitating condensation reactions under aqueous conditions.

Recent studies have expanded the role of clay minerals beyond simple adsorption and catalysis, emphasizing the importance of nanoconfined environments. For example, Bezaly et al. [39] demonstrated that antagonistic interactions between amino acids with different affinities for clay surfaces can simultaneously induce interlayer stabilization and partial exfoliation, leading to the formation of heterogeneous nano-environments within the same mineral phase. These include ordered interlayers as well as nanocavities formed by structural distortion, which may provide physicochemical conditions distinct from those of the bulk solution.

Such dynamically generated confined domains may enhance prebiotic reactivity by promoting molecular proximity, stabilizing intermediates, and enabling localized environments with distinct pH, hydration, or ionic strength. In this sense, clay minerals can act not only as catalytic substrates but also as generators of spatially heterogeneous microenvironments that may have been critical for early chemical evolution.

### 4.2. Other Mineral Systems and Catalytic Diversity

Beyond clay minerals, a wide variety of inorganic materials have been proposed as catalysts or reactive surfaces in prebiotic environments, including metal oxides and sulfide minerals. These systems are particularly relevant in hydrothermal contexts, where redox gradients and mineral interfaces can promote carbon fixation and other key transformations [40,41,42,43].

These examples illustrate the diversity of mineral-mediated pathways that may have contributed to prebiotic chemistry and highlight the potential of heterogeneous systems to support complex reaction networks.

### 4.3. Surface Reactivity and Activation Mechanisms

Beyond simple adsorption, mineral surfaces can influence the electronic properties of adsorbed species. Interactions with surface sites, including defects, vacancies, and coordinated metal centers, may facilitate charge-transfer processes and stabilize reactive intermediates [36,44,45]. Such effects are particularly relevant for the activation of small, otherwise inert molecules such as N_2_ and CO_2_, as discussed in the previous section. In this regard, mineral surfaces may act as heterogeneous catalysts, lowering activation barriers and enabling reaction pathways that are not accessible in homogeneous environments.

### 4.4. Microenvironments and Non-Equilibrium Conditions

Another important aspect of mineral-mediated chemistry is the generation of microenvironments that differ significantly from bulk conditions. Surface interfaces can create localized regions characterized by gradients in pH, ionic strength, hydration, and temperature [6,46]. In addition, processes such as wet–dry cycles, adsorption–desorption equilibria, and confinement within pores or interlayer spaces can further modulate chemical reactivity. These dynamic conditions are particularly relevant for prebiotic chemistry, where non-equilibrium processes are likely to have played a crucial role in driving molecular complexity [46,47].

Recent studies have also emphasized the importance of coupling mineral surfaces with external energy sources, such as ultraviolet radiation, geothermal heat, or redox gradients. These inputs can provide the energy required to overcome kinetic barriers, particularly when combined with adsorption and catalytic effects at interfaces [48]. In such systems, mineral surfaces not only concentrate reactants but also participate directly in energy transduction processes.

### 4.5. Transition Toward Phosphate-Based Systems

Within this broad framework, phosphate-based minerals occupy a particularly interesting position due to their chemical affinity with modern biological systems. Their ability to interact with organic molecules, stabilize intermediates, and participate in ion exchange processes makes them attractive candidates for mediating prebiotic reactions. Compared to other mineral systems, phosphate materials provide a compositional and structural continuity with biological chemistry that may be particularly relevant in the context of early molecular evolution.

This does not imply that phosphate-based minerals, or hydroxyapatite in particular, were necessarily more effective than all other mineral systems under prebiotic conditions. Rather, different minerals may have contributed distinct and complementary functions. Clay minerals offer high surface area, charged interlayers, and nanoconfined environments that are especially relevant for adsorption and oligomerization processes. Metal sulfides are particularly important in redox chemistry and hydrothermal scenarios, whereas silica and alumina surfaces can promote dehydration and acid–base catalysis. Borate minerals, in turn, have been widely discussed in relation to the stabilization of carbohydrate-like molecules. Within this broader mineralogical landscape, hydroxyapatite is distinctive because it combines structural phosphate groups, calcium-rich adsorption sites, chemical stability in aqueous environments, and a high capacity for ionic substitution. These features do not make hydroxyapatite universally superior, but they provide a specific combination of adsorption, molecular organization, and potential catalytic functionality that justifies its consideration as a complementary platform in prebiotic chemistry.

### 4.6. Concluding Remarks

Altogether, these observations support a view in which mineral surfaces are not passive substrates, but active and multifunctional environments that can promote adsorption, activation, and transformation of prebiotic molecules. Recent integrative approaches further reinforce the importance of mineral–organic interfaces in early chemical evolution [45,49]. This perspective provides a foundation for understanding how heterogeneous systems may have contributed to the emergence of molecular complexity and sets the stage for exploring specific materials, such as hydroxyapatite-based systems, in greater detail.

## 5. Hydroxyapatite as a Platform for Prebiotic Chemical Evolution

Calcium phosphates comprise a broad family of minerals with diverse compositions, structures, and physicochemical properties, ranging from highly soluble phases such as dicalcium phosphate to more stable and crystalline materials such as hydroxyapatite. These compounds differ in their calcium-to-phosphate ratios, hydration states, and structural organization, which in turn determine their stability and reactivity under different environmental conditions. In prebiotic contexts, poorly crystalline and non-stoichiometric calcium phosphate phases are likely to have played a central role due to their higher surface area and enhanced reactivity.

Among calcium phosphate minerals, hydroxyapatite (Ca_10_(PO_4_)_6_(OH)_2_) represents one of the most thermodynamically stable calcium phosphate phases under physiological conditions and is characterized by a hexagonal crystal structure with channels occupied by hydroxyl groups. Its lattice can accommodate a wide variety of ionic substitutions (e.g., carbonate, fluoride, and metal cations), leading to significant structural and chemical variability. This adaptability is particularly relevant in prebiotic environments, where mineral phases were unlikely to be perfectly crystalline or stoichiometric, and where defects and substitutions may have played a critical role in generating reactive surface sites [7,50,51].

Hydroxyapatite (hereafter HAp) is a structurally complex calcium phosphate mineral that occupies a unique position at the interface between inorganic chemistry and biological systems. As the principal mineral component of bones and teeth, it is intrinsically associated with biomolecular organization and stability in modern organisms. However, beyond its biological relevance, hydroxyapatite also exhibits a set of physicochemical properties that make it a compelling candidate for mediating prebiotic chemical processes.

These structural features give rise to a heterogeneous surface landscape characterized by calcium-rich domains, phosphate groups, and hydroxyl channels, all of which can participate in chemical interactions. Such structural flexibility is particularly relevant under prebiotic conditions, where non-stoichiometric and defect-rich mineral phases were likely predominant. These imperfections can generate active sites with distinct electronic and coordination properties, enabling adsorption and activation of molecular species that would otherwise remain unreactive in homogeneous environments.

A key consequence of this structural heterogeneity is its strong affinity for charged and polar molecules. This chemical heterogeneity enables hydroxyapatite to interact strongly with charged and polar molecules through both Lewis acidic and Brønsted basic surface sites, facilitating adsorption and stabilization processes at the mineral interface.

Beyond simple adsorption, hydroxyapatite surfaces can impose spatial organization on adsorbed species. The periodic arrangement of surface sites and the presence of crystallographic planes with distinct compositions can influence molecular orientation and proximity, effectively reducing the entropic cost of reaction. This effect is particularly important for condensation reactions, such as peptide bond formation, where precise alignment of functional groups is required. In this sense, hydroxyapatite does not merely concentrate reactants but also pre-organizes them in configurations that are more conducive to chemical transformation.

Recent experimental and computational studies have provided compelling evidence that hydroxyapatite can interact strongly with complex biomolecules, particularly nucleic acids. DNA has been shown to adsorb onto hydroxyapatite surfaces through interactions involving its phosphate backbone and the calcium ions exposed at the mineral interface. Notably, these interactions can preserve the secondary structure of the biomolecule under certain conditions, indicating that adsorption does not necessarily lead to denaturation. The stability of DNA on hydroxyapatite surfaces depends on factors such as surface composition, crystallographic orientation, and local charge distribution, illustrating how local surface properties modulate biomolecular interactions [11,12].

Although DNA itself was unlikely to be present during the earliest stages of prebiotic evolution, studies of DNA–hydroxyapatite interactions provide valuable model systems for understanding more general principles of mineral–organic adsorption, stabilization, and interfacial organization that may also be relevant to simpler prebiotic molecules. In addition to surface adsorption, hydroxyapatite can promote the encapsulation of biomolecules within its structure during mineral growth.

In such processes, the biomolecule itself can act as a template for nucleation, guiding the formation of calcium phosphate clusters that eventually reorganize into hydroxyapatite. This templating effect is particularly significant, as it reinforces the idea that organic molecules can influence the structure and growth of inorganic phases, leading to hybrid materials with emergent properties. Notably, the phosphate groups of DNA provide a structural motif that closely resembles the inorganic phosphate network of hydroxyapatite, facilitating this cooperative interaction.

Such encapsulation processes have important implications for prebiotic chemistry. Experimental studies have shown that DNA embedded in hydroxyapatite nanoparticles exhibits enhanced resistance to chemical degradation and enzymatic attack. This protective effect indicates that mineral phases could have served as reservoirs for fragile molecular information, shielding them from harsh environmental conditions such as ultraviolet radiation, temperature fluctuations, and chemical oxidation. While DNA itself is unlikely to have been present in early prebiotic environments, these observations support the broader concept that mineral matrices could stabilize and preserve complex organic structures, including potential precursors to informational polymers.

Another important property of hydroxyapatite is its ability to participate in the activation of small molecules through coordinatively unsaturated calcium sites and defect-induced electronic perturbations. The interaction of gases such as N_2_ and CO_2_ with mineral surfaces is typically limited by high activation barriers. However, the presence of coordinatively unsaturated calcium sites, coupled with defect-induced electronic perturbations, may facilitate the adsorption and partial activation of such molecules. When combined with external energy inputs, such as thermal gradients or ultraviolet radiation, these interactions can lead to reaction pathways that are inaccessible in bulk aqueous environments. Indeed, recent studies have demonstrated that hydroxyapatite-based systems can promote the formation of simple amino acids from gaseous precursors under relatively mild conditions [16].

In this context, recent advances in photocatalytic systems provide valuable mechanistic analogues that, although not strictly prebiotic, reinforce the role of surface-controlled reaction pathways in amino acid formation. Notably, the selective synthesis of glycine through C–N coupling between carbonyl intermediates and reduced nitrogen species has been demonstrated using engineered catalytic surfaces. A representative example involves the photocatalytic conversion of polyol-derived substrates into glycine via the formation of glycolaldehyde as a key intermediate, which subsequently reacts with NH_4_^+^/NH_3_ to yield C–N coupled products and ultimately glycine [52]. In parallel, photocatalytic studies have demonstrated that glycolaldehyde can be selectively formed and stabilized under controlled conditions, highlighting its key role as a reactive intermediate in carbon–carbon and carbon–nitrogen coupling processes [53]. These studies reinforce the broader concept that aldehyde intermediates can act as key nodes in the emergence of molecular complexity when coupled with suitable nitrogen sources. More generally, they illustrate how differentiated catalytic functionalities and controlled interfacial reactivity can promote selective C–N bond formation. Although these systems rely on engineered catalysts and optimized conditions, they provide valuable conceptual analogues supporting the idea that mineral surfaces on the early Earth may have stabilized reactive intermediates and facilitated selective reaction pathways [53].

Despite these promising features, it is important to recognize that hydroxyapatite alone may not provide sufficient catalytic activity to drive more complex transformations, such as peptide bond formation, at significant rates. While its surface can facilitate adsorption and organization, the activation of functional groups often requires stronger Lewis acid sites or more specialized catalytic centers. This limitation highlights the importance of considering hydroxyapatite not as an isolated catalytic material, but as part of a broader class of hybrid systems in which multiple functionalities are combined.

In this context, the integration of hydroxyapatite with catalytically active species, such as transition metals or metal oxides, represents a particularly attractive strategy. By coupling the organizational capabilities of hydroxyapatite with the catalytic properties of metal centers, it becomes possible to design systems in which molecular organization, catalytic activation, and chemical transformation occur in a coordinated manner. Zirconium-based materials, for example, are known to exhibit strong Lewis acidity and have been shown to catalyze amide bond formation under relatively mild conditions [17,18,19]. When incorporated into or onto hydroxyapatite structures, such species could provide the necessary catalytic functionality to complement the adsorption-driven processes of the mineral surface.

These findings collectively support a view of hydroxyapatite as a multifunctional platform for prebiotic chemistry, capable of mediating key steps in the progression from simple molecules to more complex chemical systems. Its ability to adsorb and organize reactants, interact with and stabilize biomolecules, and participate in mineral–organic hybrid systems positions it as a central component in models of surface-mediated chemical evolution. Rather than acting as a passive scaffold, hydroxyapatite emerges as an active participant in the generation of molecular complexity, particularly when considered in conjunction with external energy sources and complementary catalytic components.

This perspective provides a conceptual framework for understanding how mineral surfaces could have contributed to the emergence of early biochemical functionality. In particular, it highlights the importance of cooperative interactions between inorganic and organic components, as well as the role of heterogeneous environments in overcoming the limitations of homogeneous chemistry. In the following section, we explore in greater detail the role of catalytically active metal centers, with particular emphasis on zirconium-based systems, and their potential contribution to peptide bond formation under prebiotic conditions.

## 6. Hydroxyapatite: Structure, Surface Chemistry and Prebiotic Relevance

Hydroxyapatite (HAp, Ca_10_(PO_4_)_6_(OH)_2_) crystallizes in a hexagonal structure (space group P6_3_/m) and exhibits a high degree of structural and chemical versatility. Rather than reiterating its general relevance in prebiotic chemistry, this section focuses on the physicochemical features that govern its interfacial behavior, including structural heterogeneity, surface reactivity, and dynamic interactions with molecular species.

### 6.1. Structural Features and Compositional Flexibility

**Figure 4:**
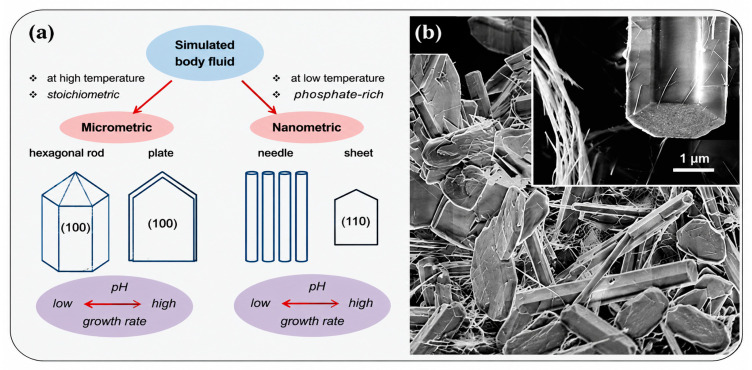
Morphological variability of hydroxyapatite (HAp) crystals under different physicochemical conditions. (**a**) Schematic representation of HAp crystal growth in simulated body fluid (BF) as a function of temperature, stoichiometry, and pH, illustrating the formation of micrometric hexagonal rods and plates at high temperature and nanometric needles and sheets at lower temperature. Crystal growth rates increase with pH, and crystallographic indices indicate the dominant growth faces. (**b**) Representative SEM micrograph showing diverse morphologies, including rod-like, plate-like, and belt-like structures obtained under controlled experimental conditions. (**a**) was reproduced from [57] with permission. (**b**) was reproduced with permission from [58]. Copyright © 2020 American Chemical Society.

The HAp lattice consists of calcium ions coordinated with phosphate groups and hydroxyl channels, forming a stable yet chemically adaptable framework. This structure can accommodate a wide range of ionic substitutions (e.g., carbonate, fluoride, silicate, and metal cations), which significantly affect crystallinity, solubility, and surface reactivity [51,54,55,56]. The structural versatility of hydroxyapatite is further reflected in its morphological variability, which is strongly dependent on environmental conditions and directly influences surface properties and reactivity [57,58] (Figure 4).

The influence of ionic substitution extends beyond structural modification and can directly affect catalytic behaviour. Recent studies have shown that the incorporation of catalytically active oxyanions such as molybdate, tungstate, and vanadate can substantially enhance the reactivity of hydroxyapatite-based materials by modifying surface acidity, adsorption properties, porosity, and redox activity. Molybdate- and tungstate-containing hydroxyapatites have demonstrated high catalytic efficiency in the selective oxidation of benzyl alcohol under mild conditions, whereas vanadate substitution has been shown to significantly enhance oxidative dehydrogenation reactions. These observations highlight the broader potential of compositional tuning as a strategy for generating reactive mineral interfaces and place zirconium-containing systems within a wider family of catalytically modified hydroxyapatites [59,60,61].

In natural environments, hydroxyapatite rarely occurs as a perfectly crystalline phase. Instead, variations in stoichiometry, crystallinity, and phase composition generate structurally diverse materials with distinct surface properties and reactivities [55]. Such variability is expected to have been particularly relevant under prebiotic conditions, where fluctuating environments favored the formation of heterogeneous and metastable mineral phases.

Under prebiotic conditions, it is unlikely that hydroxyapatite existed as a perfectly crystalline and stoichiometric phase. Instead, poorly crystalline, defect-rich, and ion-substituted forms are expected to have been predominant. Such structural imperfections introduce heterogeneity at both the bulk and surface levels, generating a variety of chemically distinct sites that may exhibit different reactivities. This compositional and structural flexibility is particularly relevant for prebiotic chemistry, where non-equilibrium conditions and variable environments would favor the formation of metastable and heterogeneous mineral phases. In addition to compositional variability, hydroxyapatite can also exhibit significant electronic and structural modifications under external stimuli. For instance, thermally induced polarization has been shown to generate long-lived internal electric fields associated with the formation of hydroxyl vacancies and charge separation within the lattice [62]. These polarized states can alter both bulk and surface properties, including electrical conductivity and charge distribution, thereby influencing the interaction with adsorbed species. From a prebiotic perspective, such electroactive behaviour may have played a role in promoting charge-transfer processes and stabilizing reactive intermediates at mineral interfaces.

### 6.2. Surface Chemistry and Reactive Sites

The surface of hydroxyapatite is characterized by the coexistence of Ca^2+^ cations, phosphate groups, and hydroxyl functionalities, resulting in a chemically heterogeneous interface capable of multiple interaction modes. A detailed description of these interaction mechanisms has been reported for hydroxyapatite-based systems [53]. Exposed calcium ions act as Lewis acid sites, while phosphate and hydroxyl groups contribute to hydrogen bonding and electrostatic interactions.

These properties enable strong interactions with a wide range of organic molecules, including amino acids and carboxylic acids, as extensively demonstrated for mineral–organic interfaces [14,35]. Surface charge is highly sensitive to environmental conditions such as pH and ionic strength, allowing dynamic modulation of adsorption behavior. Moreover, structural modifications such as polarization can induce significant changes in surface composition and organization. Experimental studies have shown that polarized hydroxyapatite surfaces may exhibit alterations in hydrogenophosphate content, changes in phosphate environments, and the formation of defect-rich regions [62]. These modifications can directly influence adsorption mechanisms and the strength of interaction with organic molecules, suggesting that surface chemistry in natural systems may have been considerably more dynamic and responsive than typically assumed for ideal mineral phases.

While adsorption phenomena are well documented for a broad range of organic and inorganic species, adsorption alone should not be interpreted as direct evidence of catalytic activation. Rather, the potential catalytic role of hydroxyapatite is generally inferred from its capacity to modify local molecular environments, alter interaction patterns, and facilitate surface-mediated reaction pathways. Although a variety of catalytic effects have been reported for hydroxyapatite-based materials, the detailed molecular mechanisms responsible for bond activation remain only partially understood in many cases.

Furthermore, defects, vacancies, and substitutions introduce additional heterogeneity, generating high-energy sites that can modify adsorption behavior, surface charge distribution, and local reactivity [35,44]. Although these features are often associated with enhanced catalytic performance, direct quantitative correlations between defect density and catalytic efficiency in specific prebiotic reactions remain scarce. Nevertheless, such structural heterogeneity is widely considered a plausible source of increased surface reactivity and catalytic versatility. The catalytic capabilities of hydroxyapatite-based systems can be further enhanced by coupling electron-transfer properties with photoactivation mechanisms. For instance, hydroxyapatite/ZrO_2_ nanocomposites exhibit a synergistic effect in which ZrO_2_ promotes N_2_ activation via π-backdonation, while hydroxyapatite provides electron-rich binding sites that facilitate subsequent reduction steps, leading to efficient NH_3_ production under mild conditions [63].

### 6.3. Adsorption and Molecular Organization

Building on the surface chemistry described above, hydroxyapatite also exhibits a strong capacity to concentrate and organize organic molecules at the interface. Experimental studies have shown that mineral surfaces can significantly increase local concentrations of reactants, overcoming dilution constraints and promoting molecular interactions [8,14]. In addition, adsorption onto mineral surfaces can impose specific orientations and reduce molecular mobility, thereby increasing the probability of productive reactions.

The adsorption behaviour of hydroxyapatite is strongly influenced by physicochemical parameters such as specific surface area, crystallinity, particle size, morphology, and synthesis route. Experimental studies have shown that variations in these characteristics can significantly affect adsorption capacity, adsorption kinetics, and the strength of interaction with both organic and inorganic species [64,65]. Such factors further contribute to the diversity of interfacial environments that hydroxyapatite may provide under prebiotic conditions.

Hydroxyapatite surfaces may also act as templates for molecular organization, facilitating the alignment of functional groups required for condensation reactions such as peptide bond formation. Although adsorption, molecular concentration, and orientational effects have been experimentally demonstrated for a variety of molecules on hydroxyapatite surfaces, direct quantitative evidence linking these phenomena to enhanced peptide yields under prebiotic conditions remains limited. Consequently, the proposed role of molecular pre-organization should currently be regarded as a mechanistically plausible hypothesis supported by indirect experimental observations rather than as a fully established reaction mechanism. Moreover, nanostructured hydroxyapatite, including porous or defect-rich systems, can generate confined environments that differ from bulk solution conditions, potentially enhancing reaction rates and stabilizing intermediates. The versatility of permanently polarized hydroxyapatite is also evident in its ability to catalyze CO_2_ conversion into a wide range of oxygenated organic compounds under mild conditions. Continuous-flow studies have demonstrated the efficient production of formic acid, acetic acid, and ethanol, highlighting the potential of these materials as sustainable catalysts for carbon fixation and carbon–carbon bond formation [66]. More broadly, these findings support the capacity of hydroxyapatite-based systems to generate reactive carbon intermediates relevant to subsequent carbon–nitrogen coupling and prebiotic synthesis pathways. In addition, nanostructured and non-ideal hydroxyapatite systems can present complex structural features, including amorphous layers, secondary phosphate phases, and nanoscale heterogeneities [67]. These characteristics generate confined interfacial environments with locally distinct physicochemical conditions, which may increase the probability of productive molecular encounters, reduce diffusion limitations, and support multiple reaction pathways simultaneously. Such effects are particularly relevant for condensation and coupling reactions, where local concentration and molecular organization strongly influence reaction efficiency.

### 6.4. Formation Pathways and Implications for Early Earth Environments

Hydroxyapatite can form through a variety of aqueous processes, including direct precipitation from calcium- and phosphate-containing solutions. Its formation is highly sensitive to pH, temperature, ionic composition, and the presence of additives or organic molecules [51,55].

Simple precipitation reactions can initially yield amorphous calcium phosphate, which subsequently transforms into more stable crystalline phases such as hydroxyapatite. Variations in synthesis conditions strongly influence particle size, morphology, and surface properties, as widely demonstrated in controlled experimental systems including precipitation, sol–gel, and template-assisted approaches [51,54,55].

In addition, template-directed and self-assembly processes can generate nanostructured hydroxyapatite with high surface area and tunable porosity, enhancing its adsorption capacity and reactivity. Such processes may have analogues in prebiotic environments, where fluctuating physicochemical conditions and organic–mineral interactions could have guided mineral formation. Processes such as polarization or interaction with charged species can promote the emergence of hierarchical structures and phase coexistence, which in turn influence the reactivity and catalytic potential of the material [67]. These observations suggest that hydroxyapatite on the early Earth may have exhibited a wide range of structural states depending on local geochemical conditions.

### 6.5. Interaction with Biomolecules and Structural Preservation

Hydroxyapatite is known to interact strongly with complex biomolecules, particularly nucleic acids. DNA adsorption onto hydroxyapatite surfaces occurs primarily through interactions between the phosphate backbone of the biomolecule and calcium ions exposed at the mineral interface. Significantly, such interactions can preserve the secondary structure of the biomolecule under certain conditions, indicating that adsorption does not necessarily lead to denaturation.

In addition to surface adsorption, hydroxyapatite can incorporate biomolecules within its structure during mineral growth. In such cases, the organic molecule can act as a template for nucleation, guiding the formation of calcium phosphate clusters that subsequently reorganize into crystalline or semi-crystalline phases. This process leads to the formation of hybrid organic–inorganic materials with emergent structural and functional properties.

Although DNA itself is unlikely to have been present in early prebiotic environments, these observations support the broader concept that mineral matrices could stabilize and protect complex organic molecules or their precursors. Such stabilization may have been essential in protecting labile species from degradation under harsh environmental conditions.

### 6.6. Prebiotic Relevance and Functional Implications

The combination of structural heterogeneity, defect formation, and electroactive behavior is consistent with the idea that hydroxyapatite may have actively influenced interfacial reactivity under prebiotic conditions. Instead, it likely acted as a dynamic interface capable of modulating charge distribution, influencing reaction pathways, and participating in cooperative catalytic processes. Taken together, the structural, surface, and adsorption properties of hydroxyapatite support its potential role as an active interface in prebiotic chemistry. Its ability to concentrate, stabilize, and organize organic molecules suggests that it could have contributed to the emergence of molecular complexity on early Earth.

Recent technological developments further support the ability of permanently polarized hydroxyapatite-based systems to directly promote the synthesis of amino acids under mild conditions. Notably, processes involving mixtures of CO_2_, CH_4_ and N_2_ in the presence of water and catalysts based on permanently polarized hydroxyapatite and brushite phases have been reported to yield glycine and alanine [68]. These systems operate under relatively low temperatures and pressures, reinforcing the role of electroactive calcium phosphate materials as active platforms for simultaneous carbon and nitrogen fixation.

Beyond amino acids, permanently polarized hydroxyapatite has also been demonstrated as an efficient catalyst for C–N bond formation through the synthesis of urea from CO_2_ and NH_3_ under relatively mild conditions [69,70,71].

These combined physicochemical features suggest that hydroxyapatite may have acted as a dynamic and functionally active interface in prebiotic environments. For instance, hydroxyapatite may have facilitated the concentration of small molecules, stabilization of reactive intermediates, promotion of condensation reactions under aqueous conditions, and spatial organization of molecular assemblies.

Additionally, the intrinsic relationship between calcium phosphate minerals and phosphorus chemistry provides a direct link to biologically relevant processes. This reinforces the hypothesis that phosphate minerals could have acted as key platforms for prebiotic reactions.

Although most studies on hydroxyapatite have been conducted in biomedical or materials science contexts, the fundamental physicochemical principles governing its behavior are directly transferable to early Earth scenarios. Integrating these insights into prebiotic chemistry frameworks offers a more comprehensive understanding of how mineral–organic interfaces may have contributed to the origin of life.

### 6.7. Transition Toward Hybrid Catalytic Systems

The integration of hydroxyapatite with catalytically active species represents a promising strategy to overcome these limitations. By combining the adsorption and organizational capabilities of hydroxyapatite with the catalytic properties of metal centers, it becomes possible to design systems in which molecular concentration, activation, and transformation occur in a coordinated manner.

In this context, the incorporation of transition metals or metal oxides into hydroxyapatite structures may enhance their catalytic performance and enable reaction pathways that are otherwise inaccessible. Such hybrid systems provide a conceptual bridge between simple mineral surfaces and more complex catalytic environments, and may offer valuable insights into the mechanisms underlying prebiotic condensation reactions.

This perspective sets the stage for exploring the role of specific catalytic elements, such as zirconium, in promoting peptide bond formation under prebiotic conditions.

## 7. Catalytic Role of Metal Centers in Prebiotic Condensation Reactions

Metal centers are widely recognized as key components in catalysis, owing to their ability to coordinate, activate, and transform molecular substrates through a variety of electronic and structural mechanisms [17,72]. In the context of prebiotic chemistry, metal ions and metal-containing minerals may have played a central role in facilitating reactions that are otherwise kinetically hindered under ambient conditions.

One of the fundamental features of metal-mediated catalysis is the capacity of metal centers to act as Lewis acids, coordinating electron-rich functional groups and thereby polarizing chemical bonds. This interaction can lower activation barriers and stabilize reactive intermediates, enabling transformations such as condensation, hydrolysis, and redox reactions. For example, the formation of amide bonds, which is essential for peptide synthesis, can be significantly enhanced in the presence of metal ions capable of coordinating both carboxylate and amine functionalities [73,74,75].

Beyond classical Lewis acid behavior, transition metal chemistry provides important insight into how small and chemically stable molecules can become activated at catalytic interfaces. In particular, studies on dinitrogen activation have shown that metal centers are capable of promoting electronic redistribution within the N_2_ molecule, facilitating subsequent transformation and functionalization steps [25,76,77,78]. It is worth noting that these processes are not restricted to complete reduction to ammonia, but may also contribute to direct nitrogen incorporation into organic frameworks through C–N bond formation pathways.

These findings have important implications for prebiotic chemistry. Although most studies on dinitrogen activation have been conducted in homogeneous systems, the underlying principles of coordination, electron transfer, and bond polarization may also be relevant to heterogeneous mineral surfaces [44,79,80]. In this context, metal sites embedded within or supported on mineral matrices could mimic key aspects of molecular catalysts, providing localized environments in which small molecules such as N_2_, CO_2_, or simple carbonyl compounds become activated toward further transformation.

The ability of metal centers to stabilize specific intermediates is particularly relevant for complex reaction networks involving multiple steps. For example, the formation of amino acids may proceed through intermediate species such as aldehydes or imines, whose reactivity can be modulated by coordination to metal ions [81,82,83]. In such scenarios, the metal center does not merely accelerate a single reaction step, but rather orchestrates a sequence of transformations by selectively stabilizing key intermediates along the reaction pathway.

In addition, recent advances in catalytic systems have highlighted the importance of cooperative and multifunctional active sites, where distinct electronic roles are distributed across different components of the catalyst [25,84,85,86]. Such systems can combine redox activity, acid–base functionality, and structural organization, leading to enhanced selectivity and efficiency. This concept is particularly relevant in the context of mineral-based prebiotic chemistry, where heterogeneous surfaces naturally present a diversity of chemically distinct sites.

Within mineral systems, the presence of transition metals or metal ions associated with phosphate or oxide matrices can introduce catalytic functionalities that are not present in purely inorganic frameworks. These metal centers may originate from geochemical processes, including weathering, hydrothermal activity, or incorporation into mineral lattices. Their distribution is likely to be heterogeneous, resulting in a spectrum of catalytic sites with varying coordination environments and reactivities [42,87,88].

Importantly, the combination of adsorption phenomena with metal-mediated activation provides a powerful mechanism for overcoming the limitations of homogeneous prebiotic chemistry. While adsorption concentrates and organizes reactants at the surface, metal centers can facilitate bond activation and transformation, enabling reaction pathways that are otherwise inaccessible in dilute aqueous environments. This synergy between physical confinement and chemical activation is a defining feature of heterogeneous catalytic systems.

Collectively, these considerations support the view that metal centers, whether present as isolated ions, clusters, or components of mineral phases, could have played a critical role in prebiotic chemical evolution. By enabling the activation of inert molecules, stabilizing reactive intermediates, and promoting bond-forming reactions such as amide formation, metal-mediated processes provide a plausible route toward increasing molecular complexity under early Earth conditions [35,37,50]. These insights lay the groundwork for exploring specific metal-containing systems, such as zirconium-based materials, which may exhibit particularly favorable properties for peptide bond formation.

## 8. Zirconium-Containing Systems and Peptide Bond Formation

Zirconium-containing materials have attracted increasing attention as highly effective catalysts in a wide range of condensation reactions, owing to their strong Lewis acidity, high coordination flexibility, and stability under diverse chemical conditions. These properties make zirconium-based systems particularly relevant in the context of prebiotic chemistry, where robust and multifunctional catalytic environments are required to promote reactions under mild and heterogeneous conditions.

Recent studies have further demonstrated that zirconium-based metal–organic frameworks (MOFs), particularly robust porous architectures such as the University of Oslo (UiO) series and MOF-808, are highly effective heterogeneous catalysts for direct amide bond formation under relatively mild conditions [89]. These systems exploit the presence of coordinatively unsaturated Zr^4+^ sites, which act as strong Lewis acid centers capable of activating carboxylic acid functionalities toward nucleophilic attack by amines.

Although such highly engineered framework materials are unlikely to have existed in prebiotic environments, they provide valuable mechanistic model systems because their active sites and catalytic pathways can be characterized by exceptional precision. Consequently, their relevance in the present context lies primarily in the catalytic principles they reveal—particularly regarding Lewis acid activation, substrate organization, and cooperative reaction mechanisms—rather than in their direct geological plausibility.

Notably, the catalytic performance of these materials has been shown to depend not only on the intrinsic acidity of the metal nodes but also on structural parameters such as linker functionality, pore size, and particle size. Among these, ultra-small Zr-MOF nanoparticles exhibit enhanced catalytic activity due to increased accessibility of active sites and improved diffusion of reactants within the porous framework. These findings reinforce the relevance of zirconium-based materials as informative models for understanding catalytic pathways that may facilitate peptide bond formation under prebiotic conditions, especially in heterogeneous and confined environments.

From a coordination chemistry perspective, Zr(IV) centers exhibit a strong affinity for oxygen-donor ligands, such as carboxylates and phosphates, enabling the formation of stable yet reactive coordination complexes. This feature is especially important for prebiotic condensation reactions, including peptide bond formation, where simultaneous activation of both carboxyl and amino functionalities is required. By coordinating to carboxylate groups, zirconium centers can increase the electrophilicity of the carbonyl carbon, thereby facilitating nucleophilic attack by amine groups and lowering the activation barrier for amide bond formation.

**Figure 5:**
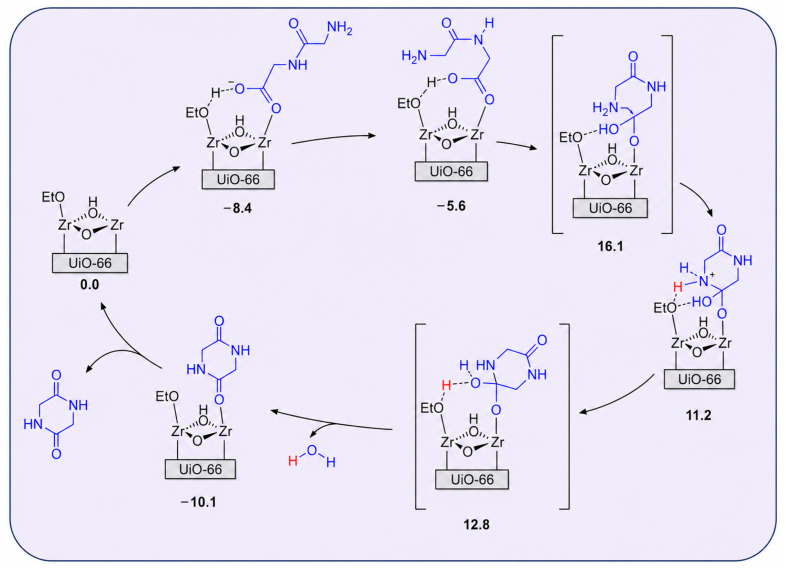
Proposed mechanism for peptide bond formation catalyzed by Zr_6_-oxo cluster-based metal–organic frameworks. The mechanism involves coordination of the carboxylate group to a Zr(IV) center, nucleophilic attack by the amine, and proton-transfer steps assisted by adjacent Zr-bound ligands, leading to cyclization and water release. Adapted from de Azambuja et al. [19].

Recent experimental studies provide direct support for this mechanistic picture. Notably, zirconium-based metal–organic frameworks (Zr-MOFs) incorporating Zr_6_-oxo clusters have been shown to catalyze peptide bond formation under mild and aqueous-compatible conditions [19]. These systems enable direct condensation of amino acid derivatives without the need for activating agents, highlighting the intrinsic catalytic capability of Zr(IV) centers. Detailed kinetic and computational analyses indicate that the reaction proceeds through Lewis acid activation of the carboxylate group, followed by nucleophilic attack and proton-transfer steps facilitated by adjacent Zr-bound alkoxy ligands [74,90,91]. Notably, the mechanism favors direct cyclization pathways rather than proceeding through ester intermediates, reinforcing the efficiency of Zr-centered activation in promoting amide bond formation [19]. This cooperative catalytic mechanism, involving Lewis acid activation and ligand-assisted proton transfer, is illustrated in Figure 5.

Furthermore, the cooperative nature of Zr_6_ clusters, in which multiple metal centers can participate in substrate activation and proton shuttling, provides a level of catalytic complexity that resembles heterogeneous mineral surfaces. This type of multimetallic cooperation has been widely recognized as a key factor in enhancing catalytic efficiency in condensation reactions [91,92]. In addition, the remarkable structural robustness and water tolerance of Zr-MOFs distinguish them from many conventional Lewis acid catalysts, which are typically deactivated under aqueous conditions [19].

In heterogeneous systems, zirconium species may be present as isolated ions, clusters, or incorporated within extended frameworks such as oxides or hybrid mineral phases. In all these cases, the catalytic behavior of zirconium is strongly influenced by its local coordination environment, including the presence of defects, vacancies, and neighboring functional groups. Such structural diversity can generate a distribution of active sites with different reactivities, which may be advantageous for promoting multistep reaction pathways [44,86]. Representative zirconium-based catalytic systems relevant to amide and peptide bond formation are summarized in Table 1.

Recent advances in transition metal chemistry have highlighted the importance of coordinatively flexible metal centers in promoting transformations of chemically stable small molecules under mild conditions. Rather than acting solely as isolated catalytic sites, metal centers can generate cooperative interfacial environments in which adsorption, electronic redistribution, and bond activation occur in a coupled manner [25]. Although zirconium is not traditionally regarded as a classical catalyst for dinitrogen reduction, zirconium-containing systems exhibit strong Lewis acidity and structural versatility, properties that may facilitate the activation and functionalization of small molecules at mineral–organic interfaces.

In this context, it is conceivable that zirconium-containing surfaces or hybrid materials could contribute indirectly to nitrogen activation processes by stabilizing reactive intermediates or facilitating C–N bond formation steps following initial activation events. Rather than acting as primary sites for N_2_ reduction, zirconium centers may play a complementary role in downstream transformations, such as the incorporation of nitrogen into organic molecules or the coupling of pre-activated species.

A key aspect of zirconium-based catalysis is its ability to operate within multifunctional systems, where different catalytic roles are distributed across distinct sites. Recent catalytic studies have emphasized the importance of such cooperative effects, in which Lewis acidic metal centers, redox-active components, and structural features act in concert to promote selective transformations [25]. This concept is particularly relevant for mineral-based prebiotic chemistry, where heterogeneous surfaces naturally provide a diversity of chemical environments.

In addition to these cooperative effects, recent studies on discrete zirconium oxo clusters have revealed that catalytic activity can also emerge from highly dynamic coordination environments at the cluster surface [93]. In particular, mechanistic and kinetic analyses demonstrate that Zr_6_O_8_ clusters can simultaneously coordinate both carboxylic acid and amine substrates, with ligand exchange processes and substrate binding occurring in a dynamic equilibrium at the cluster interface. This adaptive coordination environment facilitates the formation of catalytically active configurations in which both reactants are positioned in close proximity, enabling efficient C–N bond formation.

Recent advances in defect-engineered zirconium-based metal–organic frameworks provide a particularly illustrative example of such multifunctional catalytic systems. It has been shown that the deliberate introduction of structural defects in MOFs can enable the spatial association of Lewis acidic Zr sites with additional functional groups, such as hydrogen-bond donors or acceptors, within the same local environment [96]. In these systems, catalytic activity arises from the cooperative interaction between these distinct functionalities, which together facilitate substrate activation and stabilize key intermediates during amide bond formation. Notably, density functional theory studies indicate that hydrogen-bonding networks formed at defect sites can significantly lower activation barriers, highlighting the importance of microenvironment engineering in heterogeneous catalysis. These observations suggest that catalytic activity cannot always be attributed exclusively to zirconium centers themselves, but often emerges from the cooperative interplay between Lewis acidic metal sites, structural defects, hydrogen-bonding networks, and local confinement effects. These findings closely resemble enzymatic active sites, where multiple functionalities act in concert, and reinforce the idea that structurally complex mineral-based systems could have provided similar cooperative catalytic environments under prebiotic conditions.

In this context, metal–organic frameworks provide an especially relevant platform, as their modular architecture allows the incorporation of multiple metal centers and functional groups within a single structure [97]. BTC-based MOFs, derived from benzene-1,3,5-tricarboxylate (BTC) linkers, for example, exhibit high surface areas, tunable pore environments, and well-dispersed active sites, which collectively enhance catalytic efficiency and selectivity. The coexistence of different catalytic functionalities within these frameworks enables cooperative effects, where substrate adsorption, activation, and transformation can occur in a coordinated manner. Such features are particularly attractive from a prebiotic perspective, where heterogeneous and multifunctional catalytic systems are likely to have played a key role in early chemical evolution.

Zirconium-containing materials are also known for their ability to catalyze amide bond formation under relatively mild conditions, including in aqueous or partially hydrated environments. This is of particular importance for prebiotic chemistry, as peptide bond formation in water is both thermodynamically and kinetically challenging. The presence of zirconium centers capable of coordinating and activating reactants may therefore provide a viable pathway for overcoming these limitations.

Recent mechanistic studies have further clarified the nature of the active catalytic species in such systems, showing that zirconium salts can rapidly generate oxo-cluster species under reaction conditions, which act as the true catalysts in direct amidation processes [94]. These multinuclear clusters enable cooperative activation of carboxylic acids and amines, lowering activation barriers and allowing efficient amide bond formation under relatively mild and less restrictive conditions. These findings also highlight the importance of considering metal speciation under reaction conditions, as the catalytically active forms may differ significantly from the initial precursors.

More generally, extensive studies on catalytic amidation reactions highlight the intrinsic challenges associated with direct condensation between carboxylic acids and amines, particularly under mild conditions. The formation of stable ammonium carboxylate salts and the unfavorable thermodynamics in aqueous environments often require either high temperatures or the use of dehydrating agents to drive the reaction forward [98]. Catalytic strategies have therefore focused on facilitating both activation of the carboxylic acid and efficient water removal, as well as on stabilizing key intermediates along the reaction pathway. These considerations are highly relevant in prebiotic contexts, where the absence of sophisticated activating agents would necessitate alternative mechanisms—such as surface-mediated processes or multifunctional catalytic sites—to overcome these intrinsic limitations.

In this context, recent studies have highlighted that the catalytic performance of zirconium-based systems is strongly influenced by the structural organization of active sites and the accessibility of coordination environments within the material. For instance, zirconium-containing frameworks and cluster-based systems exhibit tunable reactivity depending on ligand exchange dynamics, substrate diffusion, and the spatial arrangement of catalytic centers. These factors determine not only the efficiency of amide bond formation but also the tolerance of the system to water and competing interactions, which are critical parameters under prebiotic conditions.

Remarkably, zirconium oxo clusters have been shown to catalyze direct amide bond formation from non-activated carboxylic acids and amines without the need for anhydrous conditions or water scavengers, highlighting their intrinsic robustness under protic environments [95,99]. Experimental studies indicate that these systems can operate efficiently in solvents such as ethanol while maintaining catalytic activity despite the presence of water generated during the reaction. This behaviour is particularly relevant in prebiotic scenarios, where strict exclusion of water is unrealistic, and supports the idea that certain mineral-like catalytic systems could overcome thermodynamic and kinetic constraints through cooperative surface interactions and dynamic ligand exchange mechanisms.

Furthermore, when combined with mineral supports such as hydroxyapatite, zirconium species can give rise to hybrid systems in which adsorption, organization, and catalytic activation occur simultaneously. In such systems, the mineral surface can concentrate and orient reactants, while zirconium centers provide the necessary catalytic functionality for bond formation. This synergy between surface-mediated processes and metal-centered catalysis represents a powerful mechanism for enhancing reaction efficiency under prebiotic conditions.

The potential relevance of zirconium in prebiotic chemistry is also supported by its capacity to form stable oxide and phosphate phases with pronounced Lewis acidic character. Nevertheless, zirconium was almost certainly less abundant than major geochemical elements such as iron, magnesium, or calcium in most early Earth environments. Therefore, zirconium is not proposed here as a globally dominant prebiotic catalyst, but rather as a mechanistically informative example of how localized mineral phases containing strong Lewis acidic centers could cooperate with adsorptive mineral substrates to promote bond-forming reactions. In this context, zirconium-based systems provide valuable insight into catalytic principles that may be transferable to a broader range of mineral–organic interfaces.

Furthermore, recent developments highlight that the catalytic behaviour of zirconium-based systems can be modulated through factors such as defect density, linker functionalization, and particle size, which directly affect the accessibility and reactivity of active sites [89]. In a prebiotic context, such structural sensitivity indicates that even subtle variations in local mineral composition and formation conditions could have led to significant differences in catalytic performance. These well-defined molecular and framework-based catalytic systems provide a mechanistic reference that can be extended to more complex and heterogeneous environments, such as mineral–metal hybrid interfaces.

**Figure 6:**
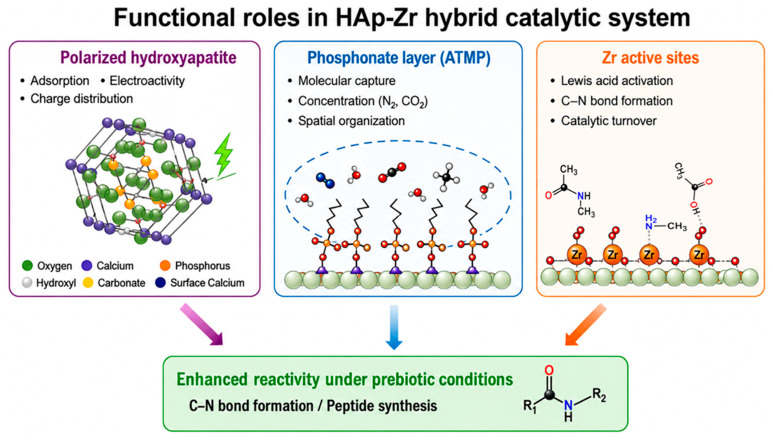
Functional roles of the components in hydroxyapatite–zirconium hybrid catalytic systems. Hydroxyapatite provides an electroactive and heterogeneous surface that promotes adsorption and charge distribution. Phosphonate ligands (ATMP) introduce molecular capture and spatial organization, enhancing the local concentration of small molecules. Zirconium centers act as coordination-active sites with Lewis acidic character, enabling substrate activation and facilitating C–N bond formation. The cooperative interaction between these components gives rise to an integrated catalytic interface in which adsorption, activation, and organization are functionally coupled, leading to enhanced reactivity under prebiotic conditions.

Overall, these observations indicate that zirconium-containing systems may serve as informative mechanistic models for understanding key processes in prebiotic chemical evolution, particularly those related to condensation reactions and peptide bond formation. By combining strong Lewis acidity, structural versatility, and compatibility with mineral matrices, zirconium-based materials represent promising candidates for bridging the gap between simple prebiotic molecules and more complex biomolecular structures. The functional roles of these components within hybrid catalytic systems can be conceptually distinguished, as illustrated in Figure 6.

## 9. Hybrid Hydroxyapatite–Zirconium Systems as Bifunctional Prebiotic Catalysts

Hybrid systems combining hydroxyapatite (HAp) and zirconium-containing phases represent a particularly compelling class of materials for prebiotic chemistry, as they integrate complementary functionalities within a single catalytic platform. While hydroxyapatite provides an efficient matrix for adsorption, molecular organization, and phosphate-mediated interactions, zirconium centers introduce strong Lewis acidity and catalytic activity. The synergy between these components enables the emergence of bifunctional systems in which molecular concentration, activation, and transformation occur in a coordinated and spatially organized manner, a concept widely recognized in heterogeneous catalysis and surface-mediated reactivity [17,44,86].

A key aspect of such hybrid systems is that hydroxyapatite does not act as a passive support, but rather as an active component whose physicochemical properties can be significantly enhanced through structural modification. For example, thermally stimulated polarization of HAp has been shown to induce the formation of OH vacancies, increase crystallinity, and enhance electrical conductivity, leading to long-lasting electroactive behaviour [62]. These structural changes are associated with the generation of internal electric fields and surface charge distributions that can strongly influence adsorption processes and interfacial reactivity. In addition, polarization modifies the surface chemistry of the mineral, including changes in hydrogenophosphate content and phosphate environments, together with the formation of defect-rich regions, all of which may influence interactions with organic and inorganic species.

Although permanently polarized hydroxyapatite exhibits distinctive physicochemical properties under experimental conditions, its occurrence and long-term persistence in early Earth environments remain uncertain. In the present context, polarized hydroxyapatite is therefore considered primarily as a mechanistic model illustrating how electroactive mineral surfaces and internal electric fields may influence adsorption, molecular activation, and catalytic behavior.

**Figure 7:**
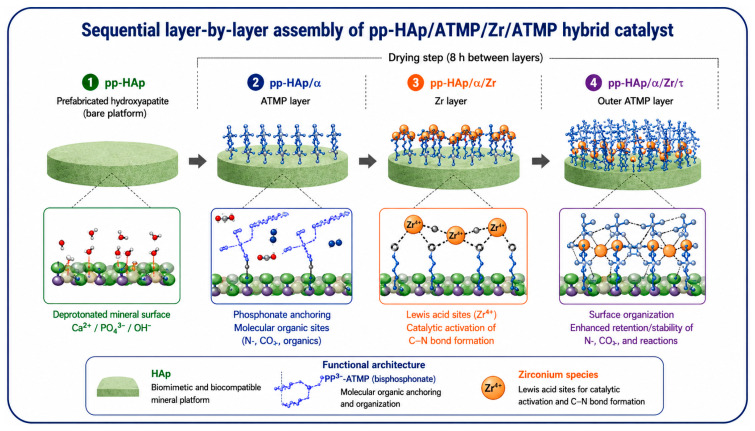
Sequential layer-by-layer assembly of the pp-HAp/ATMP/Zr/ATMP hybrid catalyst. The polarized hydroxyapatite (pp-HAp) surface (1) acts as an electroactive and heterogeneous platform that supports subsequent functionalization. The initial deposition of aminotris(methylenephosphonic acid) (ATMP) (2) introduces phosphonate anchoring groups that promote molecular adsorption and concentration. The incorporation of zirconium species (3) generates Lewis acid active sites capable of activating substrates and facilitating C–N bond formation. A final ATMP layer (4) enhances surface organization and contributes to the formation of structured interfacial domains. This hierarchical architecture integrates adsorption, catalytic activation, and spatial organization within a single bifunctional platform relevant to surface-mediated prebiotic chemistry.

The preparation of representative hydroxyapatite-based hybrid catalysts has been described using a sequential layer-by-layer deposition approach [99]. The preparation of these hybrid catalysts is based on a sequential layer-by-layer strategy, in which permanently polarized hydroxyapatite disks are coated with aminotris(methylenephosphonic acid), zirconyl chloride, and a second aminophosphonate layer. This procedure generates pp-HAp/ATMP/ZC/ATMP architectures in which each component contributes differently to adsorption, activation, and interfacial organization. A schematic illustration of this process is shown in Figure 7.

In these systems, permanently polarized hydroxyapatite (pp-HAp) acts as the substrate and is sequentially coated with aminotris(methylenephosphonic acid) (ATMP) and zirconium precursors, typically zirconyl chloride, forming a three-layer architecture (pp-HAp/ATMP/Zr/ATMP). Each component plays a distinct role: hydroxyapatite contributes to charge transfer and electrochemical activation, ATMP facilitates adsorption and organization of small molecules such as N_2_, and zirconium species act as Lewis acid catalytic centers capable of promoting bond activation processes. This cooperative arrangement enables the emergence of multifunctional catalytic behavior at the mineral interface.

**Figure 8:**
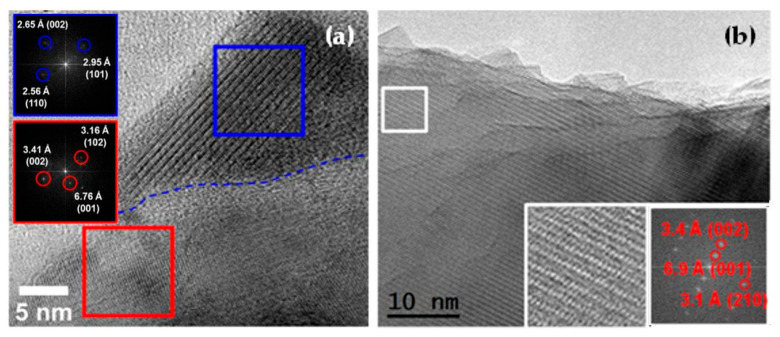
High-resolution structural characterization of hydroxyapatite-based catalytic systems. (**a**) HRTEM image of a hydroxyapatite/zirconia nanocomposite highlighting the intimate interface between both phases, where zirconium oxide domains exhibit distinct lattice spacings and are in close contact with the hydroxyapatite surface. This interfacial arrangement supports the cooperative mechanism in which zirconium species promote dinitrogen activation, while hydroxyapatite provides adsorption sites and facilitates charge transfer and stabilization of reactive intermediates. (**b**) HRTEM image of permanently polarized hydroxyapatite showing well-defined lattice fringes and the presence of a characteristic superstructure associated with specific crystallographic planes, which has been related to enhanced electronic properties and catalytic activity. (**a**) was reproduced from [66] with permission. (**b**) was reproduced with permission from [67]. Copyright © 2020 American Chemical Society.

Recent mechanistic studies on hydroxyapatite–zirconium nanocomposites have provided direct experimental evidence for this functional division (Figure 8a) [66]. Notabl, zirconium oxide domains have been shown to promote the initial activation of dinitrogen through π-backdonation mechanisms, while the activated species subsequently migrate and bind to calcium sites on the hydroxyapatite surface. This spillover-like process highlights the complementary roles of both components, with zirconium acting primarily as an activation center and hydroxyapatite as a platform for adsorption, stabilization, and further transformation of reactive intermediates. This behavior is fully consistent with the catalytic architecture described above for layer-by-layer assembled systems, where phosphonate groups contribute to molecular organization, zirconium centers to bond activation, and polarized hydroxyapatite to charge transfer and interfacial stabilization.

Further structural investigations have revealed that permanently polarized hydroxyapatite exhibits complex surface features, including the presence of superstructures, amorphous surface layers, and secondary calcium phosphate phases such as β-tricalcium phosphate [67,68]. High-resolution transmission electron microscopy studies have revealed the presence of a characteristic superstructure in permanently polarized hydroxyapatite, with lattice periodicities associated with specific crystallographic planes (Figure 8b) [67]. This superstructural organization has been linked to enhanced electronic properties, including improved charge transport and stabilization of polarized states, which are considered key factors underlying the catalytic activity of the material [67]. The observed structural motifs introduce additional heterogeneity and create a distribution of reactive sites with different coordination environments. Such heterogeneity is expected to play a crucial role in prebiotic chemistry, where non-uniform and defect-rich materials may provide a broader range of catalytic possibilities compared to ideal crystalline systems. The role of structural heterogeneity and defect-driven reactivity has been extensively documented in catalytic materials [81,86].

When integrated with zirconium-containing phases, permanently polarized hydroxyapatite systems evolve into structurally organized catalytic interfaces in which different surface functionalities operate cooperatively. Such multifunctional arrangements are well recognized in heterogeneous catalysis, where adsorption, activation, and transformation steps are spatially coupled to enhance reaction efficiency [17,100]. Experimental studies on multi-component assemblies composed of polarized HAp, zirconia, and aminophosphonate ligands have shown that phosphonate groups promote molecular organization and local concentration effects, while zirconium centers provide catalytic activation and hydroxyapatite contributes to interfacial charge redistribution and structural stabilization [101]. Together, these cooperative interactions generate catalytic environments capable of integrating multiple reaction steps within the same interfacial framework.

This division of roles closely resembles the organization of enzymatic systems, in which substrate binding, activation, and transformation occur at different sites within a structured environment. Notably, mechanistic studies indicate that nitrogen fixation in such hybrid systems proceeds through associative pathways that are thermodynamically more favorable under mild conditions, avoiding the high-energy barriers associated with direct bond cleavage [68,101]. These interfacial environments are analogous to catalytic microdomains observed in both mineral systems and enzymatic active sites [102,103].

**Figure 9:**
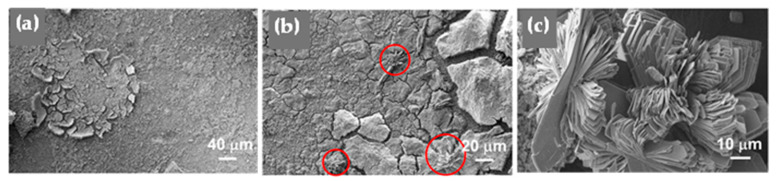
Representative SEM images showing the evolution of surface morphology in hydroxyapatite-based hybrid catalysts: (**a**) initial nucleation of surface domains, (**b**) intermediate growth and formation of zirconium-rich regions (circles show hierarchical supramolecular structures with a flower-like morphology), and (**c**) development of hierarchical flower-like supramolecular structures. These features arise from the cooperative interaction between ATMP and zirconium species and are associated with enhanced surface organization and catalytic functionality. Reproduced from [99] under the terms of the Creative Commons CC-BY 4.0 license.

The structural organization of these hybrid catalysts is also of critical importance. Detailed analyses of layer-by-layer assemblies composed of hydroxyapatite, zirconium precursors, and phosphonate ligands have shown that the formation of hierarchical supramolecular structures at the surface strongly influences catalytic performance [62]. Representative scanning electron microscopy images reveal the formation of hierarchical supramolecular architectures, including microplate assemblies and flower-like structures arising from the cooperative interaction between ATMP and zirconium species (Figure 9). In particular, the controlled deposition of multiple layers can lead to the emergence of three-dimensional architectures characterized by increased surface area, enhanced accessibility of active sites, and improved stability. These structures arise from the interplay between nucleation processes, intermolecular interactions, and surface heterogeneity, leading to complex morphologies with enhanced catalytic potential.

Such hierarchical organization introduces an additional level of functional complexity, as it allows the coexistence of multiple microenvironments within the same material. For example, regions enriched in phosphonate groups may preferentially adsorb and concentrate small molecules, while zirconium-rich domains provide catalytic activation, and hydroxyapatite interfaces facilitate charge redistribution and structural stabilization. The spatial proximity of these domains enables efficient coupling between adsorption and reaction steps, reducing diffusion limitations and enhancing overall catalytic efficiency.

Another important feature of these systems is their ability to operate under aqueous or partially hydrated conditions, which are essential for prebiotic plausibility. The combination of adsorption, confinement, and cooperative catalysis can help overcome the thermodynamic and kinetic barriers associated with condensation reactions in water. In particular, the presence of structured interfacial environments may facilitate local dehydration processes, stabilize reactive intermediates, and promote bond formation reactions that are otherwise unfavorable in bulk solution. Such constraints are well known in amide bond formation chemistry and have motivated the development of catalytic strategies capable of operating in aqueous media [77,86].

From a prebiotic perspective, these hybrid hydroxyapatite–zirconium systems provide a realistic model for multifunctional catalytic interfaces. Although the exact synthetic architectures described in modern studies may not have existed on the early Earth, analogous systems could have arisen through natural processes such as mineral precipitation, ion adsorption, and surface modification by organic molecules. The key functional elements—structural heterogeneity, cooperative catalysis, and dynamic surface interactions—are all consistent with plausible geochemical scenarios. Similar concepts have been proposed in the context of prebiotic systems chemistry and mineral-mediated evolution [37,47,89].

The overall picture emerging from these studies points to the possibility that hybrid mineral–metal systems could have played a central role in prebiotic chemistry. By integrating adsorption, activation, and transformation processes within a single platform, hydroxyapatite–zirconium systems offer a powerful framework for understanding how simple molecules may have evolved toward greater chemical complexity under early Earth conditions.

Although C–N bond formation and peptide-related chemistry constitute the primary focus of the present discussion, the multifunctional nature of hybrid hydroxyapatite–zirconium systems may be relevant to a broader range of prebiotically important transformations. The coexistence of adsorption, molecular organization, Lewis acid activation, and charge-transfer processes could potentially facilitate phosphorylation-related reactions, carbon–carbon bond formation, and redox transformations involving small carbon- and nitrogen-containing molecules. While such possibilities remain largely unexplored, they reinforce the view of these systems as potential contributors to emerging protometabolic networks rather than catalysts restricted to a single reaction class.

The combined evidence from experimental studies and recent technological developments indicates that permanently polarized hydroxyapatite-based systems constitute versatile catalytic platforms capable of promoting carbon fixation, nitrogen activation and C–N bond formation under mild conditions. These systems enable the synthesis of a wide spectrum of molecules ranging from simple C1–C3 organics to amino acids and urea [49,66,68,69,70], supporting a unified framework in which mineral surfaces actively drive chemical complexity. Such multifunctionality reinforces the potential relevance of electroactive calcium phosphate minerals in prebiotic chemistry and early metabolic evolution.

## 10. Peptide Bond Formation on Mineral Surfaces

Peptide bond formation under prebiotic conditions remains challenging because condensation reactions between amino acids are both thermodynamically and kinetically unfavorable in aqueous environments. Water elimination competes with hydrolysis processes, while the formation of stable ammonium carboxylate salts further limits direct amide bond formation under mild conditions [104,105].

To address these limitations, mineral surfaces have been widely proposed as catalytic and organizational platforms capable of promoting peptide bond formation. By providing interfacial environments where reactants are concentrated and spatially constrained, these systems enhance the probability of productive interactions and facilitate bond formation processes that are inefficient in bulk solution [106,107]. A comparative overview of the main mineral systems investigated in prebiotic peptide formation and their catalytic roles is provided in Table 2.

Although no single mineral system can be considered universally superior for all prebiotic processes, hydroxyapatite presents several distinctive features that justify its consideration alongside more extensively studied materials such as clay minerals, silica, and metal sulfides. Unlike most mineral surfaces, hydroxyapatite combines structural phosphate groups with a high capacity for ionic substitution, enabling substantial modulation of surface chemistry while maintaining structural integrity. The presence of calcium-rich surface sites promotes strong interactions with amino acids, carboxylates, phosphates, and other biologically relevant molecules, facilitating both adsorption and molecular organization. In addition, hydroxyapatite exhibits remarkable chemical stability under aqueous conditions and shares compositional similarities with modern biological phosphate-based systems. These characteristics suggest that hydroxyapatite may provide a particularly suitable interface for coupling adsorption, molecular concentration, catalytic activation, and molecular assembly within the same material. Rather than replacing other proposed prebiotic minerals, hydroxyapatite should be viewed as a complementary platform offering a unique combination of structural, chemical, and functional properties.

Although several studies indicate that hydroxyapatite can promote amino acid adsorption, concentration, and surface organization, direct quantitative comparisons of peptide yields obtained in the presence and absence of hydroxyapatite under identical prebiotic conditions remain relatively scarce. Consequently, the potential contribution of hydroxyapatite to peptide synthesis should currently be interpreted primarily in terms of its demonstrated capacity to modify the local physicochemical environment rather than as definitive evidence of universally enhanced peptide production.

Experimental studies have demonstrated that different classes of mineral systems can promote amino acid condensation. For example, silica and alumina surfaces have been shown to facilitate peptide bond formation through surface-mediated dehydration processes, particularly under thermal or fluctuating conditions [14,110]. Similarly, metal-containing minerals such as copper, zinc, or iron oxides can enhance reactivity by coordinating to carboxylate groups and activating them toward nucleophilic attack [109,111].

In addition to catalytic activation, confinement effects at mineral interfaces play a critical role. Nanoporous materials and layered structures create microenvironments with reduced water activity and altered dielectric properties, favoring condensation reactions and stabilizing transition states [112,113].

Environmental dynamics also contribute significantly to peptide synthesis. Wet–dry cycles, for instance, can drive ester-mediated pathways in which activated intermediates subsequently react with amines to form amide bonds [108,114].

The role of metal ions as Lewis acid catalysts further enhances the plausibility of mineral-mediated peptide synthesis. Coordination to metal centers can facilitate activation of the carboxyl group and stabilization of transition states, and similar mechanisms may operate in heterogeneous systems where catalytic sites are immobilized on mineral surfaces [115,116]. Recent mechanistic studies have shown that zirconium-based systems can form oxo-cluster species under reaction conditions, which act as highly active catalytic centers for direct amide bond formation [94]. These multinuclear species enable cooperative activation of reactants, providing a plausible pathway for peptide bond formation under relatively mild and partially hydrated conditions.

Compared to alternative prebiotic scenarios, mineral-mediated peptide formation offers a unique combination of catalytic efficiency and environmental relevance. While high-energy processes or chemical activating agents can enhance reaction rates, they may lack geological plausibility or continuity. In contrast, mineral surfaces provide persistent, spatially organized environments capable of sustaining chemical transformations over extended timescales [49,117].

Collectively, these findings support the view that mineral surfaces could have played a central role in overcoming the intrinsic limitations of peptide bond formation under prebiotic conditions. By integrating adsorption, catalytic activation, confinement, and environmental dynamics, mineral interfaces provide a coherent framework for the emergence of peptide-like molecules and early protometabolic systems.

## 11. Dynamic Regimes and Non-Equilibrium Conditions in Surface- Mediated Synthesis

Prebiotic environments were inherently dynamic, characterized by continuous fluctuations in temperature, hydration, and chemical composition. Rather than representing a limitation, these variations likely played a central role in driving chemical evolution by maintaining systems away from equilibrium and enabling reaction pathways that would otherwise be inaccessible under static conditions [118,119].

Gradients in temperature, concentration, redox potential, and hydration state are particularly important in this context. Such gradients can generate directional fluxes of matter and energy, leading to localized accumulation of reactants and promoting selective reaction pathways [120,121]. For example, thermal gradients in porous mineral systems or gas–liquid interfaces can drive concentration effects and selective accumulation of molecules, thereby enhancing reaction rates and promoting molecular organization. These processes highlight the importance of heterogeneous environments in sustaining chemical activity over extended timescales. The main environmental regimes relevant to surface-mediated prebiotic synthesis and their effects are summarized in Table 3.

Cyclic processes such as wet–dry transitions provide an additional mechanism for enhancing chemical reactivity. During drying phases, increased solute concentration and reduced water activity favor condensation reactions, while rehydration allows redistribution and further transformation of products [122,123].

Beyond simple cycling, non-equilibrium conditions can also introduce kinetic effects that influence chemical selection. Recent theoretical and experimental studies have shown that oscillatory regimes can lead to resonance-like behavior, where reaction rates are maximized at specific cycle frequencies. This dynamic coupling between environmental fluctuations and reaction kinetics may provide a mechanism for selecting particular reaction pathways or products, contributing to the emergence of chemical complexity.

Another important feature of non-equilibrium systems is the presence of continuous or intermittent feeding of reactants, which maintains the system in an open state. In such systems, the balance between input fluxes, reaction rates, and dissipation processes determines the overall behavior of the chemical network. This can give rise to steady states that are dynamically maintained rather than thermodynamically stable, as well as to transient regimes in which specific intermediates accumulate. These properties are characteristic of dissipative systems and are considered essential for the emergence of protometabolic networks [120,121].

Dehydration and rehydration processes are particularly relevant in surface-mediated synthesis, as they directly affect both thermodynamics and kinetics. Dry phases promote condensation reactions by reducing water activity, while wet phases facilitate diffusion and reorganization of reactants. In mineral systems, these processes may be further modulated by adsorption–desorption equilibria, confinement within pores, and surface charge effects, leading to highly dynamic and spatially heterogeneous reaction environments. Such coupling between surface chemistry and environmental dynamics may have been critical for enabling complex reaction sequences under prebiotic conditions. In this context, it is noteworthy that zirconium-based catalytic systems have been shown to operate efficiently without the need for strictly anhydrous conditions, as the formation of oxo-cluster species can create localized environments that favor condensation reactions even in the presence of water [96]. This behavior further supports the compatibility of such systems with dynamic and fluctuating prebiotic environments.

Significantly, non-equilibrium regimes do not merely accelerate existing reactions but can fundamentally alter reaction networks. Under fluctuating conditions, product distributions may be governed by kinetic accessibility rather than thermodynamic stability, allowing the persistence of otherwise unstable intermediates. This shift from thermodynamic to kinetic control is a defining feature of prebiotic systems and may have facilitated the emergence of increasingly complex molecular assemblies [122,123].

Overall, these dynamic regimes suggest that prebiotic chemistry was governed not only by the intrinsic properties of molecules and materials but also by the temporal and spatial variability of the environment. Such conditions provide a framework in which mineral surfaces, fluctuating inputs, and evolving chemical networks can interact to generate increasing levels of complexity.

## 12. Possible Implications for Early Peptide Formation and Protometabolism

The formation of peptides under prebiotic conditions has important implications for the emergence of functional chemical systems and the transition toward protometabolism. Even short peptides can exhibit catalytic, structural, and binding properties, suggesting that their early formation could have contributed to the development of primitive reaction networks. Notably, the appearance of oligopeptides capable of interacting with metal ions or mineral surfaces may have introduced new levels of chemical organization and reactivity, bridging the gap between simple organic molecules and more complex biochemical systems [124,125].

**Figure 10:**
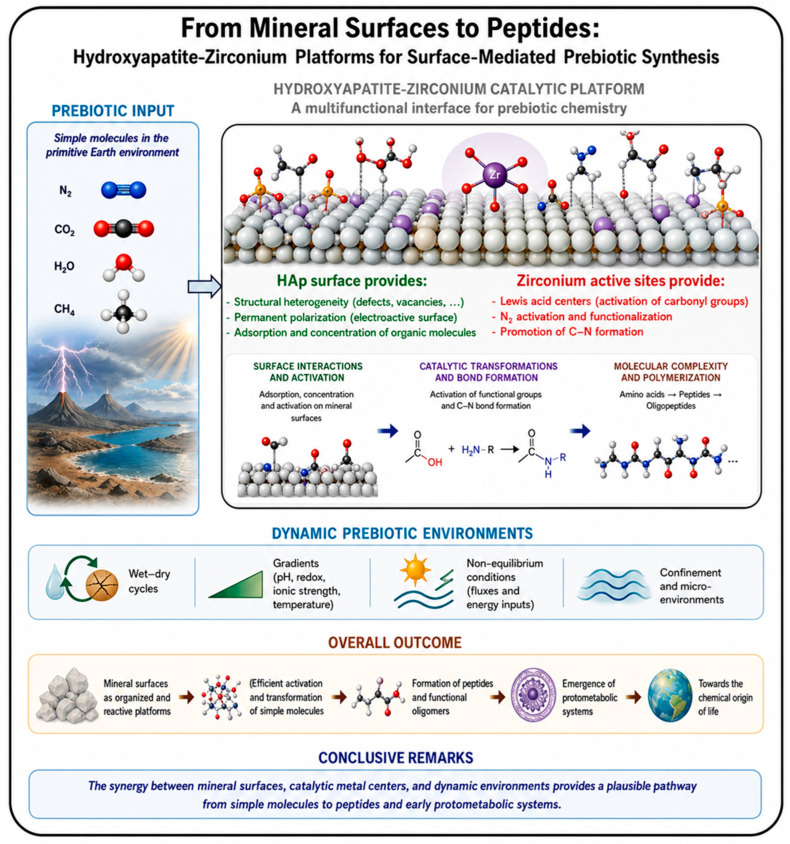
Integrated framework illustrating the role of mineral surfaces in prebiotic peptide formation. Permanently polarized hydroxyapatite (HAp) acts as an electroactive and structurally heterogeneous platform that promotes adsorption, molecular organization, and charge-transfer processes. When combined with zirconium-based species, the system evolves into a multifunctional catalytic interface in which adsorption (HAp/phosphonates), activation (Zr active sites), and spatial organization are functionally coupled. Dynamic environmental regimes, including wet–dry cycles, gradients, and non-equilibrium conditions, further enhance reactivity by enabling concentration, activation, and transformation processes. These combined effects provide a unified pathway linking simple surface-mediated reactions to the formation of amino acids, peptides, and increasingly complex protometabolic systems.

From an evolutionary perspective, peptide formation on mineral surfaces may represent a key step in the transition from prebiotic chemistry to protometabolic networks. This conceptual framework is summarized in Figure 10, which integrates the roles of mineral surfaces, catalytic systems, and dynamic environmental conditions in the progression from simple molecules to peptide-like structures and increased chemical complexity.

Rather than isolated reactions, early chemical systems are increasingly viewed as interconnected networks capable of sustaining fluxes of matter and energy. In this context, peptides could have acted as functional components within these networks, enhancing reaction rates, stabilizing intermediates, or facilitating molecular recognition processes. Such systems would not yet be biological in the modern sense but could exhibit properties associated with early forms of chemical selection and organization.

The concept of protometabolism provides a useful framework for understanding these processes. Protometabolic systems are thought to consist of networks of chemical reactions driven by environmental inputs and mediated by mineral surfaces, metal ions, or simple organic molecules. These systems are typically far from equilibrium and rely on continuous energy dissipation to maintain their structure and function. In such environments, peptides formed on mineral surfaces could become integrated into reaction networks, contributing to feedback mechanisms and increasing overall system complexity.

The role of phosphate and phosphate-containing minerals is particularly relevant in this context. Phosphorus is a key element in modern biological systems, forming the backbone of nucleic acids, contributing to energy transfer through molecules such as ATP, and participating in membrane structure. However, its availability under prebiotic conditions remains a major challenge, often referred to as the “phosphate problem.” Mineral phases such as apatite may have acted both as reservoirs and as reactive surfaces, enabling the concentration and transformation of phosphorus species under specific environmental conditions.

Phosphate minerals, including hydroxyapatite, may have played a dual role in early chemical evolution. On the one hand, they could have provided surfaces for adsorption and organization of organic molecules, facilitating condensation reactions such as peptide formation. On the other hand, their intrinsic chemical properties may have contributed to phosphorylation processes and energy transfer reactions, which are central to metabolic systems. This dual functionality makes phosphate-based minerals particularly attractive candidates for mediating the transition from simple chemistry to more complex, functionally integrated systems [126,127].

In addition, the interaction between peptides and phosphate-containing surfaces may have contributed to the emergence of selective processes. Certain amino acid sequences may exhibit stronger affinity for specific mineral surfaces or metal ions, leading to preferential stabilization and accumulation. Such interactions could introduce a primitive form of selection based on physicochemical compatibility, potentially influencing the composition and function of early peptide populations. This idea is consistent with models in which early peptides and nucleotides co-evolved within shared chemical environments, giving rise to increasingly complex molecular systems.

Importantly, the integration of peptide formation, mineral surfaces, and dynamic environmental conditions support the idea that early chemical evolution was not driven by a single dominant pathway, but rather by the interplay of multiple processes operating simultaneously. Surface-mediated synthesis, non-equilibrium dynamics, and the emergence of functional molecules such as peptides may have collectively contributed to the development of protometabolic systems capable of sustaining chemical complexity over time.

From a geochemical perspective, the potential coexistence of phosphate-based minerals such as hydroxyapatite and zirconium-bearing phases in igneous and hydrothermal environments further supports the plausibility of hybrid catalytic systems under prebiotic conditions. Zirconium is commonly found in the form of zircon (ZrSiO_4_) in igneous rocks, where it can persist through geological processes due to its high chemical stability. At the same time, phosphate minerals may form through fluid–rock interactions and hydrothermal alteration processes, leading to localized environments in which both components could be present. Such settings may have provided natural platforms for the emergence of cooperative mineral–metal catalytic systems. These geological associations suggest that the coupling between mineral surfaces and zirconium-based catalytic species may not only be chemically viable but also geochemically realistic.

The combined evidence supports a scenario in which mineral surfaces—particularly phosphate-based systems—played a central role in enabling the formation, stabilization, and functional integration of peptides in early Earth environments. Through their ability to mediate adsorption, catalysis, and chemical organization, these surfaces may have provided a bridge between prebiotic chemistry and the emergence of primitive metabolic networks.

## 13. Conclusions and Future Perspectives

The study of prebiotic chemistry has progressively shifted from the analysis of isolated reactions toward the understanding of complex, heterogeneous, and dynamic systems. Within this evolving framework, mineral surfaces have emerged as key components in early chemical evolution, capable of promoting adsorption, catalysis, and molecular organization. These properties are particularly relevant for addressing fundamental challenges such as the formation of peptide bonds under aqueous conditions and the emergence of functionally integrated chemical networks.

Among the wide variety of minerals investigated, phosphate-based systems—and hydroxyapatite in particular—have received comparatively limited attention in prebiotic chemistry, despite their intrinsic relevance to modern biological systems. The structural versatility, compositional flexibility, and surface reactivity of hydroxyapatite suggest that it could have played a more significant role in early Earth environments than is currently recognized. As discussed throughout this work, hydroxyapatite is not merely a passive scaffold but a dynamic material capable of modulating adsorption, stabilizing intermediates, and influencing reaction pathways, especially under non-equilibrium conditions. Its ability to host defects, substitutions, and electroactive states further enhances its potential as a prebiotic interface.

In parallel, the incorporation of catalytically active metal centers introduces an additional level of functional complexity. In this context, zirconium-containing systems stand out due to their strong Lewis acidity, coordination versatility, and stability under diverse chemical conditions. Although zirconium has not traditionally been considered a central element in prebiotic scenarios, growing evidence suggests that it may play a significant catalytic role, particularly in condensation reactions such as peptide bond formation. When combined with mineral matrices such as hydroxyapatite, zirconium can contribute to the development of hybrid systems in which adsorption, activation, and transformation processes operate cooperatively within structured interfacial environments.

At the same time, it is important to recognize that zirconium was likely far less abundant than major geochemical elements such as iron, magnesium, or calcium in most early Earth environments. Consequently, zirconium is not proposed here as a globally dominant prebiotic catalyst, but rather as a mechanistically informative example of how strong Lewis acidic centers can cooperate with mineral surfaces to promote bond-forming reactions. In this sense, zirconium-containing systems provide valuable insight into catalytic principles that may be applicable to a broader range of mineral–organic interfaces.

The concept of bifunctional or multifunctional catalytic platforms is particularly significant in this context. Systems that combine surface-mediated organization with metal-centered activation can overcome key limitations associated with prebiotic synthesis, including low reactant concentrations, unfavorable thermodynamics, and high kinetic barriers. Such cooperative effects are reminiscent of enzymatic catalysis, where multiple functional elements operate in concert within a structured environment. In prebiotic contexts, mineral–metal hybrid systems may have provided analogous functionalities, enabling more efficient and selective chemical transformations, consistent with models of autocatalytic and self-sustaining chemical networks [128].

Equally important is the influence of fluctuating and non-equilibrium environments on chemical evolution. Gradients, cyclic processes, and fluctuating environments can drive systems away from equilibrium, allowing access to reaction pathways and product distributions that would otherwise be inaccessible. When coupled with surface-mediated processes, these dynamic regimes can enhance condensation reactions, promote molecular organization, and support the emergence of increasingly complex chemical networks. The integration of mineral surfaces, catalytic centers, and environmental dynamics thus represents a promising framework for understanding the transition from simple chemistry to protometabolic systems.

Looking forward, several experimental directions appear particularly promising. Greater attention should be devoted to hydroxyapatite under prebiotic conditions, especially in non-stoichiometric, defect-rich, or polarized forms, in order to better understand its catalytic and interfacial properties. Likewise, zirconium-containing systems deserve further investigation in aqueous and fluctuating environments because of their potential role in condensation reactions and C–N bond formation. Finally, hybrid mineral–metal systems integrating adsorption, activation, and confinement effects represent especially attractive models for studying cooperative catalytic mechanisms under realistic prebiotic conditions.

In addition, future research should place greater emphasis on dynamic experimental setups that incorporate gradients, wet–dry cycles, and continuous or intermittent feeding of reactants. Such approaches are essential for capturing the non-equilibrium nature of early Earth environments and for identifying emergent behaviors that cannot be observed under static conditions. The combination of experimental, computational, and systems-level approaches will be crucial for advancing this field and for establishing robust connections between prebiotic chemistry and early biochemical evolution.

Bridging the gap between mineral-mediated chemistry and protometabolism remains one of the central challenges in prebiotic chemistry. Progress in this area will require not only detailed mechanistic studies, but also integrative frameworks that consider the interplay between materials, molecules, and environment, in line with current systems chemistry approaches [128,129]. In this context, the potential role of hydroxyapatite-based systems and zirconium-containing catalysts deserves particular attention, as they offer a compelling combination of chemical plausibility and functional versatility.

The overall conceptual framework proposed in this review is summarized in Figure 10, highlighting the interplay between mineral surfaces, catalytic centers, and dynamic environmental conditions in the transition from simple molecules to peptide-like structures and early protometabolic systems.

Taken as a whole, the insights presented in this work support the view of prebiotic chemistry as an inherently heterogeneous, dynamic, and cooperative process. Reconsidering the role of underexplored materials such as hydroxyapatite, and incorporating catalytically active elements such as zirconium, may open new perspectives on the pathways that led to the emergence of life. 

## Figures and Tables

**Table 1:** Zirconium-based catalytic systems relevant to prebiotic condensation reactions and C–N bond formation.

System Type	Active Species	Key Catalytic Feature	Reaction Conditions	Mechanistic Insight	Representative References
Zr salts in solution	Zr^4+^, Zr oxo-clusters (in situ)	Strong Lewis acidity, cluster formation	Mild, partially hydrated	Multinuclear cooperative activation of carboxylic acids and amines	[93,94]
Discrete Zr oxo clusters	Zr_6_O_8_-type cores	Defined active sites	Mild conditions	Dynamic surface reorganization and catalytic activity	[95]
Zr-based MOFs (UiO-type)	Zr_6_ nodes	Accessible Lewis acid sites, tunable defects	Heterogeneous catalysis	Defect-driven reactivity and substrate activation	[19,96]
MOF-derived/porous Zr systems	Zr nodes + structural defects	High surface area, site accessibility	Mild to moderate	Structure–activity relationships in catalytic amidation	[94]
ZrO_2_/hybrid oxide systems	Surface Zr centers	Acid–base bifunctionality	Thermal or aqueous	Surface coordination and activation of reactants	[90]
Mineral–Zr hybrid systems	Dispersed Zr on mineral surfaces	Cooperative adsorption + activation	Surface-mediated, heterogeneous	Coupling of surface organization and metal-centered catalysis	[61,66]

**Table 2:** Mineral systems involved in prebiotic peptide formation and their proposed catalytic roles.

Mineral System	Composition/Active Sites	Catalytic Role	Key Processes Promoted	Representative References
Clays (e.g., montmorillonite)	Layered silicates, exchangeable cations	Adsorption, confinement, templating	Oligomerization, molecular alignment, peptide formation	[13,33,38]
Silica/Alumina	Surface hydroxyl groups, Lewis acid sites	Dehydration, acid–base catalysis	Peptide bond formation, amino acid condensation	[14,33,108]
Metal oxides (e.g., TiO_2_, Fe oxides)	Surface metal centers, hydroxyl groups	Lewis acid catalysis, redox activity	Activation of carboxyl groups, C–N bond formation	[43,44]
Sulfide minerals (e.g., FeS, NiS)	Redox-active metal sulfides	Electron transfer, catalytic surfaces	Amino acid activation, peptide formation	[40,109]
Hydrothermal mineral systems	Mixed mineral assemblages, fluid–rock interfaces	Energy gradients, catalytic surfaces	Organic synthesis, amino acid formation	[41,42]
Hydroxyapatite (HAp)	Ca^2+^, PO_4_^3−^, OH^−^, defects, polarization	Adsorption, molecular organization, surface activation	Concentration of biomolecules, condensation reactions	[7,12,16,63]

**Table 3:** Dynamic environmental regimes relevant to surface-mediated prebiotic synthesis and their effects on chemical evolution.

Dynamic Condition	Description	Effect on Reaction Systems	Relevance for Peptide Formation	Representative References
Wet–dry cycles	Alternating hydration and dehydration phases	Concentration of solutes, dehydration-driven reactions	Promotes condensation and polymerization reactions	[46,114,122]
Thermal gradients	Temperature differences in porous or hydrothermal environments	Molecular transport, accumulation, and selection	Enhances reaction rates and concentration of reactants	[41,119]
Non-equilibrium feeding	Continuous or intermittent supply of reactants	Maintains systems far from equilibrium	Sustains reaction networks and prevents equilibrium stagnation	[49,118]
Confinement effects	Reactions in pores, layers, or restricted geometries	Reduced diffusion, increased local concentration	Stabilizes intermediates and enhances reaction probability	[14,37]
Surface heterogeneity	Presence of defects, charges, and diverse active sites	Multiple reaction pathways, site-specific reactivity	Enables parallel reactions and selective pathways	[84,86]
Oscillatory/cyclic conditions	Periodic environmental fluctuations (e.g., hydration, temperature)	Kinetic selection and temporal control of reactions	Favors formation of specific products over time	[122,123]

## Data Availability

No new data were created or analyzed in this study. Data sharing is not applicable to this article.
